# Urban scaling, geography, centrality: Relation with local government structures

**DOI:** 10.1371/journal.pone.0238418

**Published:** 2020-09-04

**Authors:** Anthony F. J. van Raan

**Affiliations:** Centre for Science and Technology Studies, Leiden University, Leiden, The Netherlands; Universidade Estadual de Maringa, BRAZIL

## Abstract

We investigate socio-economic urban scaling behavior of municipalities in Denmark, the Netherlands, and in particular in Germany. Our interest is twofold. First we investigate whether, and to what extent, scaling occurs in various types of urban areas. The second important topic of research concerns the comparison of specific types of urban areas with regard to the values of the gross urban product. This is a new approach: two scaling systems are compared not only in terms of the scaling exponent, but also in terms of the differences in the gross urban product. We are specifically interested in the role of urban governance in terms of local urban government structures. Germany is our central case because it works as a natural experiment: a large number of urban areas is one-governance, but others are not. More specifically, we distinguish between cities of which the surrounding urban area belongs to the municipality of the city (kreisfreie cities), and those specific districts (Kreise) which are urban areas consisting of several municipalities. Our findings suggest that urban areas with one municipality perform better than urban areas with fragmented governance structures. We also investigate the relation between scaling of Kreise and simple measures of centrality, including the Zipf-distribution. A strong relation is found between the measured residuals of the scaling equations and the socio-economic position of cities assessed with a set of different socio-economic indicators. Given the debate on the effectiveness of municipal reform, our results may lead to challenging conclusions about the importance of one-municipality instead of multi-municipality governance in urban areas. These results are relevant for policy as they suggest that there is a benefit to unifying the governance structure of compact urban agglomerations.

## Introduction and background

Recent studies on urban metrics shows a *more than proportional* (superlinear) increase of the socio-economic performance of cities (measured by the gross urban product, GUP) in relation to population size [1–3]. This *urban scaling* relation is described by a power-law dependence of GUP on population size where in most cases the values of the exponent are between 1.10 and 1.20 (see section Materials and methods). This implies that GUP increases disproportionally with city size, each 10% increase in population size is associated with about 11.5% increase in GUP. In other words, a city twice as large (in population) as another city can be expected to have approximately a 2^1.15^ = 2.22 larger socio-economic performance (in terms of the gross urban product). This urban scaling behavior is not restricted to socio-economic variables such as GUP: it is found for human interactions in general and for knowledge production activities in cities [4–7]. Indicators representing these activities appear to scale nonlinearly with the number of inhabitants of cities and urban agglomerations. Similar scaling is found for other complex systems such as universities [8]. The basis of this scaling behavior is provided by the theory of complex, adaptive systems [9]. Networked structures reinforce nonlinearly as the system increases in size, particularly more than proportional, i.e. superlinearly, described by a power law [10].

A simple way to understand this phenomenon is by realizing that the number of nodes increases *linearly* whereas the number of links between the nodes increases *superlinearly* with the growth of a network. The nodes in the urban complex system are the inhabitants, social and cultural institutions, centers of education and research, firms, etcetera. The links between these (clustered) nodes are crucial for new developments, reinforcement of urban facilities, and innovation. Because they increase superlinearly, the socio-economic strength of the city increases more than proportional with increasing size. For an extensive discussion of the theoretical basis of urban scaling we refer to [10]. Evidently, the relation between urban scaling (which is a phenomenon at the meso-level) and dynamic processes in urban systems, for instance the concentration of business companies and professions, and particularly mobility [11] and other forms of traffic relations (processes at the micro-level) are important and could provide further understanding of scaling. Also it is shown that urban-scaling based metric provides more appropriate urban indicators than the usual per capita values of indicators [12].

The spatial units used in this paper are defined by the concept of municipality which has a central position in this study. This is in contrast with most studies on urban scaling where metropolitan statistical areas (MSAs, US) [1–3] or functional urban agglomerations (FUAs, Europe) [13, 14] are used as spatial units. We think that FUAs and MSAs are useful definitions of larger urban areas but they are not appropriate for this study because they ignore precisely what we want to investigate: the influence of local government structures, in terms of formal municipality boundaries. Also there are inconsistencies in the definition of FUAs as compared to the Eurostat data on ‘greater cities’ [15].

Municipalities are entities with a very high degree of autonomy explicitly laid down in the Constitutional Law of the countries involved. A municipality consists of one or several residential areas with a local government formed by the municipal council elected in nationally organized elections and headed by a mayor appointed by the Head of State. In most cases a specific city or town is the core of the municipality. Formally, one could see this as an ‘administrative subdivision’ in the national political government system: national level, provincial (or state) level, and municipal level. But the term ‘administrative subdivision’ sounds like a bureaucratic subdivision of, for instance, cities into neighborhoods or so. It is not an appropriate terminology for an entity that has a crucial position in the Constitutional Law. Using terminology that can be felt, and possibly works as a derogatory qualification of municipalities, and is probably related to the, in our opinion, underestimation of the role of municipalities in urban science. This paper goes exactly against this.

Municipalities may have different characteristics: they can be major cities that are the centers of larger urban agglomerations; municipalities can be parts of such urban agglomerations, for instance bordering to the municipality of the central city; or municipalities can be communities in more rural regions. There will be different histories of how municipal governance structures were implemented. Our study focuses on the current situation. Throughout this paper we use the term governance for local government in the context of public administration. Also we use the term city *only* for cities defined as municipalities and not for the entire urban agglomerations such as metropolitan areas which may consist of many independent municipalities.

The interpretation of urban scaling laws is important in the discussion on models of urban growth, structure and optimal size of cities and their urban areas [16, 17]. There are ongoing discussions in the literature on the question whether urban indicators can be determined on scaling based on size alone, or that other parameters such as interactions between cities or city history also play a role. In a study on the scaling of urban indicators in England and Wales the authors [18] used population density thresholds and commuter-flow thresholds to define ‘cities’ (which are in our terminology: multi-governance (= multi-municipalities) urban areas). It was found that most urban indicators scale linearly with ‘city’ size, regardless of the definition of the urban boundaries. Research on congestion-induced delays in ‘cities’ shows that scaling does not depend on its population only, but also on previous history [19]. These authors conclude that this strong path-dependency prohibits the existence of a simple scaling mechanism valid for all ‘cities’ and that their results also challenge the use of cross-sectional data (data for different ‘cities’) for understanding longitudinal series for individual ‘cities’. For recent work on the analysis of urban scaling in time we refer to [20]. An example of scaling in a longitudinal sense is the case of the rapidly growing new city of Almere in the Netherlands, see Supporting Information of [21].

Our interest is twofold. First we investigate whether, and to what extent, urban scaling occurs in various types of urban areas. Verifying this scaling phenomenon remains important because several authors doubt the superlinear power-law relationship (for overview on this issue see [22]). Furthermore, we find that urban scaling within a country shows considerable differences depending on geography, type of urban areas, centrality, population cutoff. It is important to find, and where possible try to explain these differences in scaling behavior. In a study on urban scaling in relation to rich (West European Union) cities and relatively poor (post-communist European) cities, the authors conclude that their results suggest that superlinear urban scaling represents a phase of economic growth rather than a universal characteristic of all cities [23].

The second important topic of research concerns the comparison of specific types of urban areas with regard to the values of the gross urban product. This is a new and little explored approach: two scaling systems are compared not only in terms of the scaling exponent, but also in terms of the differences in the gross urban product. Recent work in this context relates to scaling and differences in GUP for two groups of Indian cities a [24], and to scaling and differences in GUP for two groups of Polish cities [25]. Similar comparative analyses are scaling and differences in number of homicides for two groups of Indian cities [26], scaling and difference in the number of domestic electricity connections for two groups of Indian cities [27], and scaling and differences in number of votes in US and UK urban agglomerations for two different parties [28].

For this paper the crucial point is that urban areas, particularly a city with its suburbs, can be one municipality (*one-governance* urban area), or the city as well as its (often wealthy [29]) suburbs are all different municipalities (*multi-governance* urban area). Our previous study on the urban scaling of cities in the Netherlands [21] focused on this issue by analyzing both the scaling behavior as well as the difference in GUP values of major cities in terms of the municipality of the central city and of their urban agglomerations consisting of different municipalities. In all cases power-law exponents of around 1.15 were found. But remarkably, urban areas as one formal municipality (one-governance) appeared to have higher GUP values and thus perform better as compared to an urban agglomeration with similar population size and population density but existing of a number of municipalities (multi-governance). A recent study on the role of urban governance in five OECD countries [30] strongly supports our earlier finding. The authors conclude that ‘cities’ (in our terminology: multi-governance urban areas) with fragmented governance structures tend to have lower levels of productivity. For instance, for a given population size, an urban area (e.g., metropolitan area) with twice the number of municipalities is associated with around six percent lower productivity.

The urban scaling phenomenon is important for new insights into and policy for urban development and, particularly, for municipal reform. This may concern urban agglomerations where municipal reform means an enlargement of the municipality of the central city by discontinuation of the municipalities of the suburbs, and of smaller municipalities that are merged into one new municipality. Different from the usual focus on measuring the effect of municipal reform on *expenses*, the urban scaling phenomenon relates to the expected socio-economic *profits*. Possible effects per medium-sized city could amount to hundreds of millions of euros which means thousands of jobs per year and per urban agglomeration.

In this study we investigate several aspects of urban scaling in three European Union countries, Denmark, the Netherlands, and in particular Germany. The *basic idea* of this paper is to find empirical evidence of differences in scaling in terms of the scaling exponent but particularly in terms of the values for one-governance versus multi-governance urban areas. Germany given its size and the availability of data on both one-governance (= one municipality) as well as on multi-governance urban areas offers the best possibilities. This is precisely the core issue of our study and therefore we focus on this country. We discuss the results for Denmark and the Netherlands in S1 and S2 Appendices, respectively. Given the differences in availability of further statistical data on municipalities in the three countries, we have also carried out country-specific analyzes. This enables us to experiment with new approaches where possible given the data availability.

## Data and analytical method

In this section we give an overview of the data and methods applied in this study to measure and analyze urban scaling in the three European Union countries, Germany, Denmark, and the Netherlands. As discussed in the foregoing section the focus of this study is on Germany.

**Fig 1:**
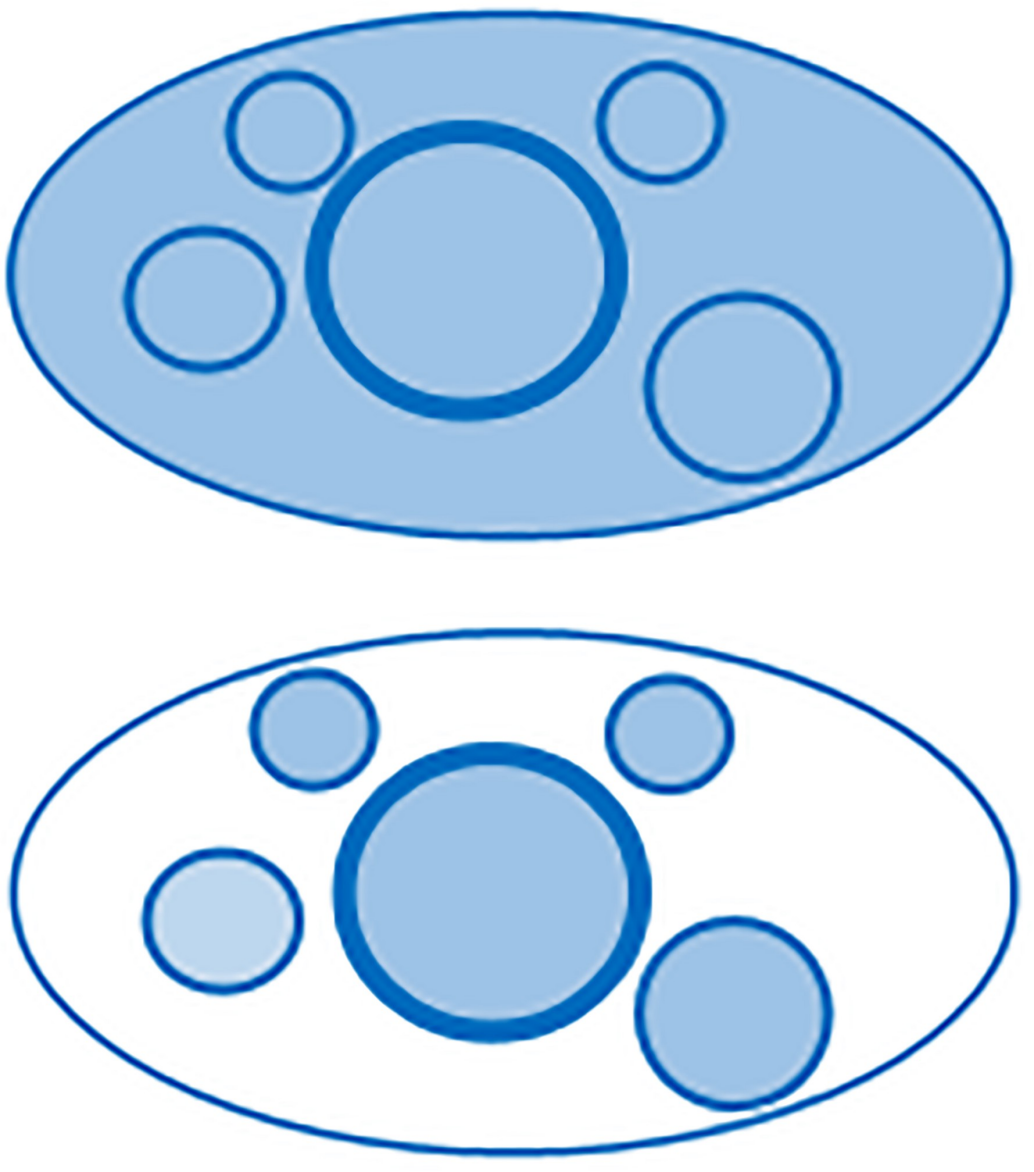
Upper part: Kreisfreie city, here the central city and its neighboring smaller cities/towns form an urban area which is *one* municipality: A mono-governance urban area. Lower part: Kreis with population size and density comparable to kreisfreie cities: central city and its neighboring smaller cities/towns form an urban area with *several different* municipalities: multi-governance urban area.

Germany with 83 million inhabitants consists of sixteen federal states. The country currently has 106 *kreisfreie* cities, and these are *examples par excellence* of the earlier discussed one-governance urban areas, i.e., cities of which the surrounding area belongs to the municipality of the city, and thus a one-governance urban area (which is in fact the definition of the concept ‘kreisfrei’). Together these cities have a population of about 27,000,000. Statistical data are also available for another important type of populated communities: the *Kreise*; these are districts around mostly smaller cities consisting of several municipalities and thus they are multi-governance structures. Together the Kreise (in total 296) have about 56,000,000 inhabitants. We illustrate the local governance structure of kreisfreie cities and Kreise with Fig 1.

For our analysis we clustered the German federal states into five major parts of the country: North Rhine-Westphalia, western part of Germany; Baden-Württemberg and Bavaria, southern part; Hesse, Rhineland-Palatinate, and Saarland, middle part; Bremen, Hamburg, Lower Saxony, and Schleswig-Holstein, northern part; Berlin, Brandenburg, Mecklenburg-Vorpommern, Saxony, Saxony-Anhalt and Thuringia, eastern part. We collected for all kreisfreie cities and all Kreise (period 1992–2014): (1) gross urban/Kreise product; (2) population (number of inhabitants); (3) surface areas; (4) population of the municipalities within Kreise [31]. We focus our analysis on the scaling behavior (scaling exponent and absolute values) of all kreisfreie cities and of all Kreise, as well specific sets of Kreise grouped by population density and by centrality.

We also investigated whether scaling is related to generative or distributive processes. The extensive amount of available data in the German case makes important further analyses possible. For instance, with a large set of socio-economic data on cities [32] we analyze the relation between these socio-economic data and specific characteristics of scaling, particularly the residuals of the empirically determined scaling equation. Moreover, the availability of data at the level of Kreise makes it possible to perform a comprehensive analysis on centrality.

The results for Denmark and the Netherlands in are discussed in S1 and S2 Appendices, respectively. In several cases results for these countries are relevant for the discussion of the findings for Germany and therefore we briefly discuss here the data used in the analysis of urban scaling in Denmark and the Netherlands. Denmark with its nearly 6 million inhabitants is administratively divided in five districts consisting of a number of municipalities. In 2007 the number of municipalities was reduced from 271 to 98 [33]. This municipal reform, however, mostly involved the smaller municipalities. Given the population size of Denmark, the number of major cities is restricted: seven municipalities have more than 100,000 inhabitants and four of these are municipalities of which the central city itself has a population of more than 100,000 (Copenhagen, Aarhus, Odense, Aalborg). And these major cities -except Copenhagen- already had municipal reforms in the 1970’s and 1980’s, only Aalborg had a municipal reform in 2007. Remarkably, so far there has been no municipality reform whatsoever of the Copenhagen agglomeration (28 municipalities). We collected the following data for all Danish municipalities (period 1993–2015): (1) gross urban product; (2) population (number of inhabitants); (3) surface areas; and (4) the population of the central city (or town in case of smaller municipalities) [34–36]. In the case of Denmark our analysis focuses on the scaling behavior (exponents and GUP values) of all municipalities and of specific sets defined by population size and by centrality. On the basis of our results, we also investigated whether scaling is related to generative or distributive processes.

The third country is the Netherlands with around 17,000,000 inhabitants and administratively divided into 12 provinces and 380 municipalities. We focus our research on one-governance versus multi-governance urban areas. To this end, we collected the following data (period 2014–2016). For 21 major cities in the Netherlands for which the Central Bureau of Statistics (CBS) has defined urban agglomerations: (1) gross urban product; (2) population (number of inhabitants); (3) surface areas; (4) population of the municipalities within the urban agglomerations [37]. These major cities and their urban agglomerations are examples of a multi-governance urban area: a centrally located city which is a separate municipality closely connected with adjacent but also separate municipalities (smaller than the central city) with a strong urban character (‘suburbs’). Also for the Netherlands we analyze the relation between socio-economic data [38] and the residuals of the scaling. Given the availability on data of well-defined urban agglomerations, we use the residuals to analyze the effect of multi-governance on gross urban product as a function of the number of municipalities in an urban agglomeration.

Our data are delivered by the national statistical offices with long standing expertise and experiences in the production of socio-economic data. There is a possibility that the definitions of GUP might slightly differ per country. Therefore, in this study no direct comparisons in absolute GUP across the three countries are made. Next to the usual statistical analysis to calculate the confidence interval of the power law exponents (see for instance [22]) we applied an additional error analyses as described in [21] and available in osf.io/4ru96.

## Results and discussion

### Geographical factors in the scaling of kreisfreie cities and Kreise

Germany is our central case because it works as a natural experiment: a large number of urban agglomerations is Kreisfrei, but others are not. The status of kreisfrei has been assigned to cities starting already in the early 19^th^ century and was primarily based on the geographic and political role of the city and less on, for instance, economic power, let alone their current scaling behavior. Indeed, as the data in this paper clearly show, there are kreisfreie cities that are relatively less powerful economically, and there are economically very strong Kreise.

**Fig 2:**
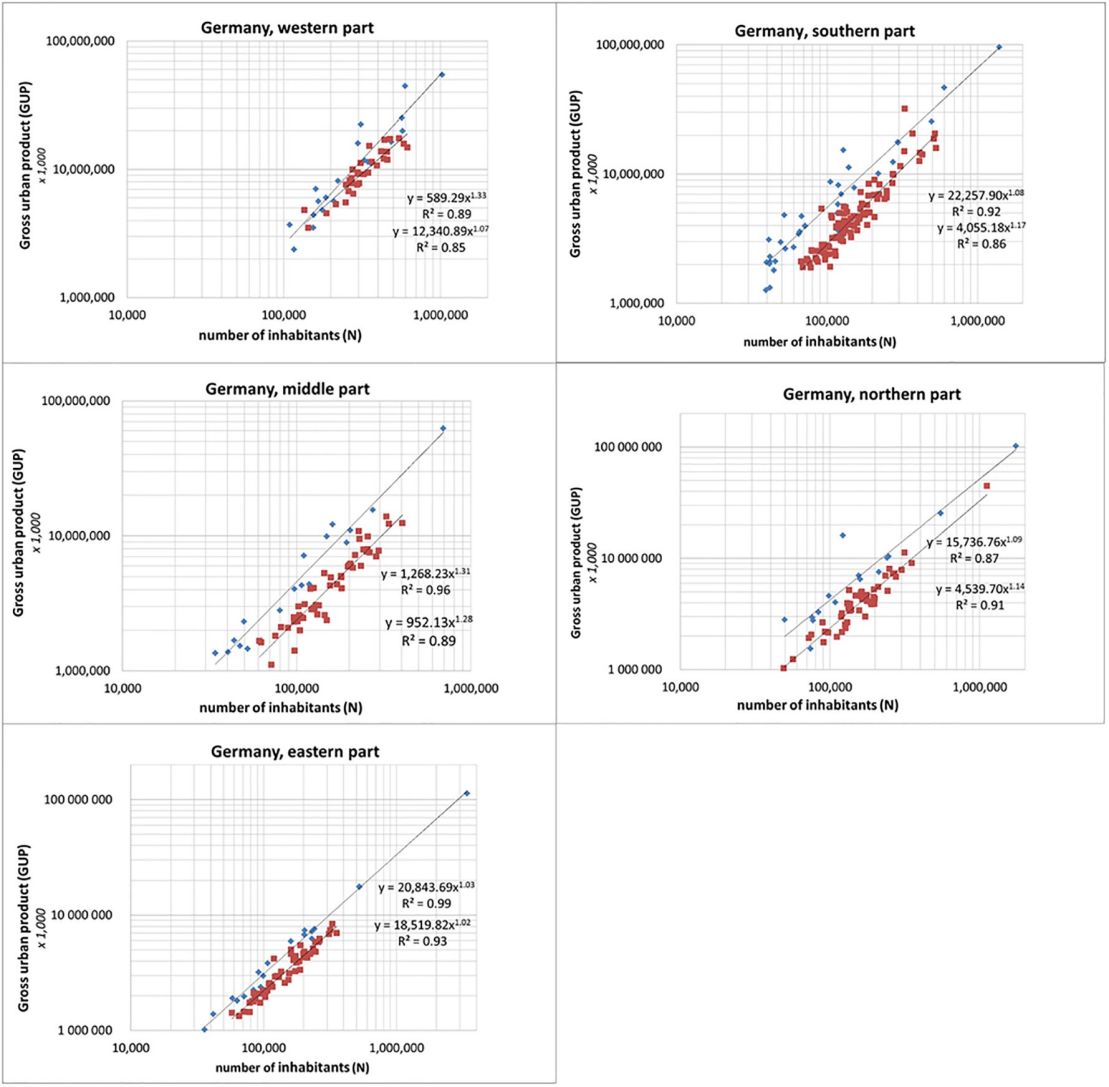
Scaling of the gross urban product (GUP) for all (kreisfreise) cities and Kreise. Upper left panel: western part of Germany; upper right panel: southern part; middle left panel: middle part; middle right panel: northern part; lower left panel: eastern part. Blue diamonds represent the (kreisfreie) cities, red squares represent Kreise (GUP in units of €1,000, data average 2012–2014).

We find that the gross urban product (GUP) scales superlinearly with population for both the kreisfreie cities (which are one-governance (= one municipality) urban agglomerations) as well as for the Kreise. As mentioned before it is important to determine the superlinearity of the scaling, but at the same time the difference in GUP values is crucial. Furthermore, we see differences between parts of the country. In Fig 2, upper left panel, the results are shown for the traditionally industrial western part of Germany. Largest city is Cologne, with a population of about 1,100,000. The kreisfreie cities scale with 1.33 (95% level of confidence and R^2^ for the scaling exponents are given in Table 1) and the Kreise with 1.07. Two cities, Düsseldorf and Bonn, take a strikingly strong position. Leaving both cities out of the analysis we find a scaling exponent of 1.25. This shows how the scaling exponent may depend on the positions of one or a few cities in the entire set. Nevertheless, the above shows that the measured scaling exponent does not change dramatically. Most notable in this comparison in scaling between Kreisfreie cities and Kreise is the clear difference in absolute GUP values. We see that, on average, these densely populated Kreise (multi-governance) underperform (in terms of GUP values) as compared to the (one-governance) kreisfreie cities.

**Table 1:** Scaling exponents for the kreisfreie cities and Kreise in the five parts of Germany. Square brackets indicate 95% CIs on scaling exponents. The number of cities and Kreise is denoted by *n*.

***cities (municip*.*)***	***n***	***GUP***	***R***^***2***^
W	22	1.33 [1.28–1.38]	0.89
S	34	1.08 [1.06–1.10]	0.92
M	17	1.31 [1.27–1.35]	0.96
N	15	1.09 [1.03–1.15]	0.87
E	19	1.03 [1.02–1.05]	0.99
***Kreise***	***n***	***GUP***	***R***^***2***^
W	31	1.07 [1.04–1.10]	0.85
S	31	1.17 [1.16–1.18]	0.86
M	51	1.28 [1.26–1.30]	0.89
N	49	1.14 [1.12–1.16]	0.91
E	57	1.02 [1.01–1.03]	0.93

Fig 2, upper right panel, shows the results of the economically most booming southern part of Germany. Largest city is Munich with a population of about 1,500,000. The kreisfreie cities scale with 1.08, and the Kreise with 1.17. Also in this scaling analysis we have a city with an extraordinary position: Ingolstadt. The GUP of this city is relatively very high because Ingolstadt (about 140,000 inhabitants) is home to the headquarters of the automobile manufacturer Audi and the headquarters of the electronic stores Media Markt and Saturn. Leaving Ingolstadt out of the analysis, the measured exponent does not change much (exponent 1.07). Also two other large car industries have their headquarters in this part of Germany: Mercedes-Benz in Stuttgart and BMW in Munich. We also see a Kreis with a very strong position: Landkreis Munich. This Kreis is the district surrounding Munich and it benefits greatly from the booming economy of Munich. If we leave this Kreis out of the analysis, the exponent for the scaling of the Kreise becomes 1.13. Looking at absolute GUP values, we again see that the Kreise underperform as compared to the kreisfreie cities.

What could be the cause of the difference in scaling exponent between the western and southern part of Germany? We think that the most plausible explanation is that in the western part only a few cities, Düsseldorf and also Bonn and Münster, have a relatively high GUP as compared to most other cities which are considerably less flourishing. In such cases the regression line becomes steeper and hence we find a larger exponent. In contrast to this situation, most of the cities in the southern part, not only the largest ones, are economically booming. As a result the regression is less steep and we have a lower exponent.

In Fig 2, middle left panel, the results for the middle part of Germany are presented. Largest city is Frankfurt with about 750,000 inhabitants. The kreisfreie cities scale with 1.31 and the Kreise with 1.28. Fig 2, middle right panel, shows the results for the northern part of Germany. Here the largest city is Hamburg with about 1,800,000 inhabitants. The kreisfreie cities scale with 1.09 and the Kreise with 1.14. In this figure we see again a city which clearly is an outlier. This city is Wolfsburg (about 125,000 inhabitants) with a very high GUP because it is the location of the Volkswagen headquarters with the world's biggest car plant, production of 815,000 cars per year (2015) and 70,000 employees in Wolfsburg alone. Measured in GUP per capita, Wolfsburg is one of the richest cities in Germany. Leaving Wolfsburg out of the analysis we find a scaling exponent 1.11. Also for these two parts of Germany we find that Kreise underperform as compared to the kreisfreie cities. But the figures also clearly show that this not necessarily the case for all individual Kreise: quite a number of Kreise overperform kreisfreie cities at a given population size. This observation is important with regard to the problem of causality: it is not very likely that Kreise are multi-governance because they are socio-economically weaker.

In Fig 2, lower left panel, the results are shown for the economically more problematic eastern part of Germany. Largest city is Berlin with a population of about 3,700,000. We see that in this part of the country scaling is hardly significant: the kreisfreie cities scale with 1.03 and the Kreise with 1.02. Nevertheless, also here kreisfreie cities overperform as compared to Kreise. In all other parts of Germany the kreisfreie cities as well as the Kreise have higher scaling exponents. These findings suggest that the mechanism behind scaling, particularly the size-based superlinear reinforcing of the socio-economic links in networked systems, works less effective in this part of Germany with its difficult economic development. A possible explanation is the decline in population and particularly the move away of talented people to other parts of Germany. Without Berlin the scaling exponent is 1.06 which is an indication that Berlin is economically underperforming. In Table 1 we present an overview of the scaling exponents for the kreisfreie cities and Kreise in the five parts of Germany.

In some cases the scaling exponent for the kreisfreie cities is larger than those of the Kreise (see the data for W, M, and E in Table 1) and in some cases we find the opposite (see the data for S and N in Table 1). This suggests that the results are inconclusive. We emphasize however that in all cases GUP is over the entire range of measurement significantly higher for kreisfreie cities than for Kreise. This is the central issue. The exponent is not the most important parameter here: as explained earlier we measure the exponent in order to determine whether superlinear scaling occurs for kreisfreie cities as well as Kreise. We find that this is the case. But the values of the measured exponents are highly dependent on one or a few individual kreisfreie cities or Kreise, as discussed above with examples.

**Fig 3:**
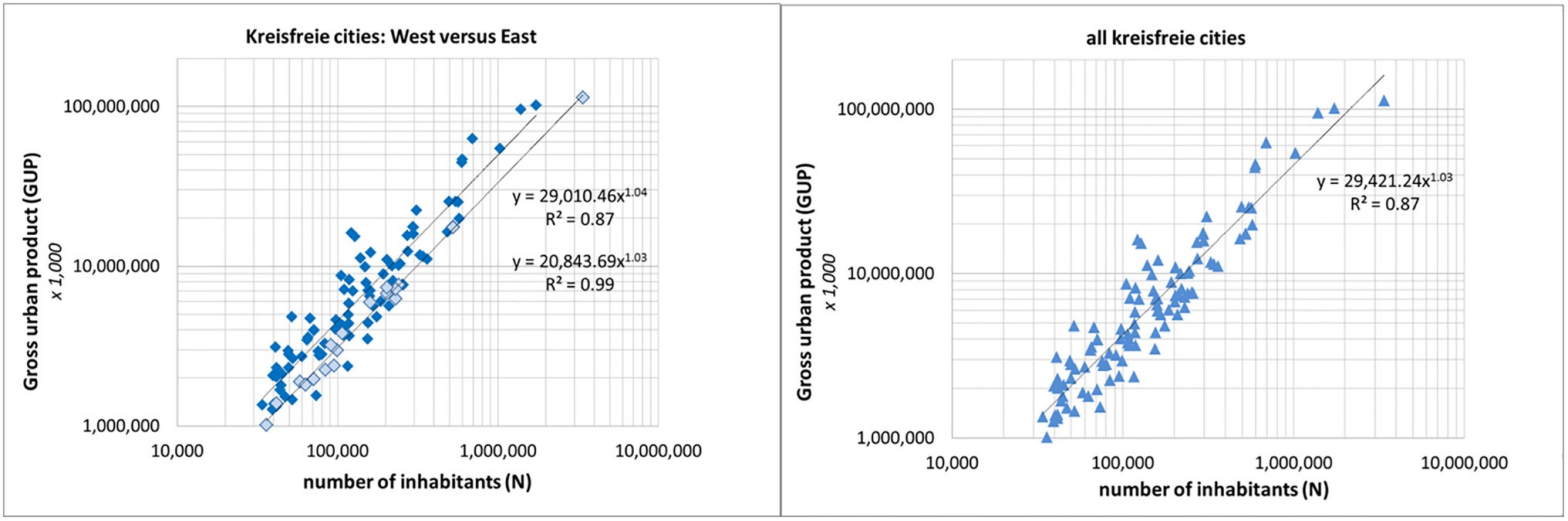
Left: Comparison of the scaling of the kreisfreie cities in West (dark blue diamonds) versus East (light blue diamonds). Right panel: scaling of all kreisfreie cities together (GUP in €1,000, data average 2012–2014).

In Fig 3, left panel, we compare the scaling of the kreisfreie cities in the former West Germany–which is the western, southern, middle and northern part- with the former East Germany. We see that the difference is not so much in the scaling exponent but in the values of socio-economic performance (GUP). This finding is supported by the household income per capita. Using for the whole of Germany the index value 1.00 we find for the west part of Germany 1.03 and for the east part 0.86 [39]. These data correspond well with the findings presented in Fig 3.

In Fig 3, right panel, the scaling of all kreisfreie cities is shown. We see that the scaling exponent is low, 1.03, lower than the scaling exponents for most parts of Germany. Without the largest city, Berlin, the scaling exponent is 1.05. This figure shows that apart from the usual statistical errors there is another source of uncertainty in the measurement of scaling in an entire country: the set of cities for which the scaling is calculated may consist of different subsets (which together form the entire set) each with their own scaling. This effect is for instance clearly present in the scaling of Polish cities where the authors show that when dividing the entire set into two subsets according to specified criteria, the scaling of the entire set differs significantly from the scaling of two subsets separately [25]. Thus the measured scaling is sensitive to the delineation of a set of specific entities (cities, Kreise). Also population cutoff used to create a set of cities will influence the measured scaling [18]. Interestingly, the measured scaling itself may suggest the existence of different power-law regimes related to population size [40].

In our study we find (see S1 Fig) that the numerous smaller kreisfreie cities in the southern part of Germany are at a high level of GUP, thus they will ‘lift’ the regression line at the lower population side thereby lowering considerably the exponent of the scaling. The largest city, Berlin, has compared to the two next largest cities, Hamburg and Munich, a relatively low GUP. This reinforces the just mentioned lowering of the scaling exponent. Thus, we find the opposite of the suggestion made in [23] that it might be possible that superlinear scaling is an effect of aggregating many cities from different locations in the same analysis. We conclude that also our research shows that one has to be careful in interpreting the empirically measured scaling behavior: although the scaling can be significantly superlinear, the value of the power law exponent will, as described above, depend on several factors, and will not necessarily be the expression of, for instance, just one prevailing characteristic of the system to which the measurements are applied.

### Effect of urban governance structure

In the foregoing section we empirically demonstrated the scaling of kreisfreie cities and Kreise with scaling exponents ranging between 1.03 and 1.33. Moreover, the figures demonstrate that in all cases the absolute values of the GUP are larger for the kreisfreie cities as compared to Kreise. In other words, kreisfreie cities generally outperform Kreise. A crucial element in our study is the question how governance structures influence socio-economic performance. Our data on kreisfreie cities and Kreise enable us to investigate this. We made the following analysis. In a number of Kreise the administrative and economic centers are cities that are not-kreisfreie cities (because they formally belong to a Kreis) although they can be considerably larger than smaller kreisfreie cities [41]. These central cities within a Kreis are separate municipalities and together with other municipalities they form a Kreis. There is an overlap in population size as well as in density between kreisfreie cities and Kreise. In such cases, Kreise are densely populated urban areas around a central city, just as the kreisfreie cities. The only difference is that the urban area of a kreisfreie city is a one-governance urban area, whereas the Kreis around a not-kreisfreie city is a multi-governance urban area.

**Fig 4:**
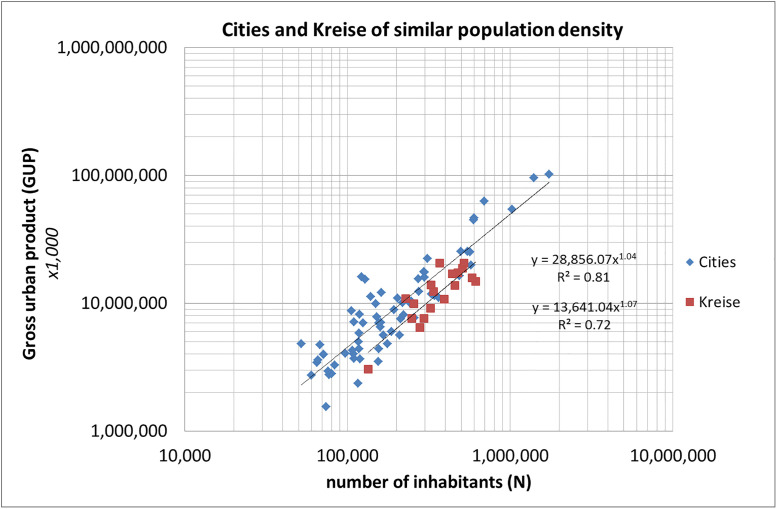
Comparison of the scaling of kreisfreie cities (blue diamonds) with Kreise (red squares) of similar population size and population density (GUP in €1,000).

We created a set of typical urban area Kreise by selecting those Kreise which have more than 50,000 inhabitants *and* density >500 inhabitants/km^2^ (the density range typical for kreisfreie cities). Then we calculated the scaling of these Kreise in comparison with the kreisfreie cities. The results are shown in Fig 4. We see that the one-municipality urban areas (the kreisfreie cities) (scaling exponent 1.04, 95%CI [1.00–1.08], R^2^ = 0.81) overperform the multi-municipality urban areas (scaling exponent 1.07, 95%CI [1.04–1.10], R^2^ = 0.72). The scaling exponent is somewhat higher for the Kreise for the given range of population size, but their values are well below those of the kreisfreie cities within the entire range of measurement and that is why the kreisfreie cities outperform the Kreise of similar population size and density. We emphasize that our observations do not allow a strict identification of causality. However, our results show that a higher GUP occurs in significantly more cases when a specific modality of local government—namely a one-municipality urban area- is present than in cases where it is absent.

An interesting issue for further research related to the causality problem is the question whether agglomerations that transitioned from multi- to mono-governance recently still look more like multi-governance agglomerations than those that underwent the transition longer ago. This could be tested by using residuals from the relevant scaling analysis. A problem is however that, for instance in Germany, transitions from multi-governance to one-governance took place mainly in the 1970’s and there are no reliable socio-economic data for that time.

### Residual analysis and comparison with socio-economic data of cities

Earlier work on urban scaling [1–4] convincingly showed that the positions of cities with respect to the scaling regression line are almost completely dominated by population size and that deviations from the average scaling equation measured by the residuals of the scaling equation show a very long temporal persistence. Effects of path dependence or spatial interactions for individual cities may therefore manifest itself in their residuals of the scaling equation. Residuals are a measure of the deviations of the *observed* (i.e., real) value from the *expected* value given by the regression line through all measuring points of a specific set (see Section “Materials and methods” for a discussion of the mathematical procedure to calculate the residuals). We calculated for all kreisfreie cities the residuals of the scaling equations. Analysis of the residuals may reveal local characteristics of individual cities in terms of success or failure relative to other cities. Positive residuals indicate that a city performs better than expected. We did not find any significant relation between population density of the kreisfreie cities and the value of the residuals.

The intriguing question now is: what about the relation between the *residuals* calculated on the basis of GUP scaling with population on the one hand, and on the other hand the socio-economic position of a city as assessed by a combination of a large number of different quantitative and qualitative indicators? The German socio-economic research agency Prognos AG carried out a study into the future perspectives of (kreisfreie) cities and Kreise on the basis of a number of indicators and published the results in the report *Zukunftatlas 2016* [32]. The Prognos method is based on 29 indicators in the fields of demographics, labor market, social welfare, competition and innovation. No scaling approaches were applied. These indicators are discussed in detail in the Prognos report [32]. These indicators were used to assess strength and dynamism of cities and urban areas. On the basis of these assessments, a ranking of all cities and Kreise was created. Particularly the maps in this report and in [42] show the division between the eastern part and the other parts of Germany in terms of the economic differences based on perceived opportunities in the near future.

By selecting the kreisfreie cities from the Prognos ranking, we find that of the *top-20 cities* 17 have a residual larger than 0.15, see Table 2, left part. For the *bottom-20 cities* we find that again 17 have a residual smaller than -0,15, see Table 2, right part. We conclude that there is a strong relation between the measured residuals in this study and the assessment of future perspectives of cities by the Prognos method. This finding relates to work on the interpretation of scaling residuals in terms of the evolution of urban functions over time, in particular urban dynamics and innovation [43, 44]. We see that not necessarily the largest cities are the most successful; medium-sized cities with leading universities and their spin-offs in science parks or with research-intensive industry are also important centers of innovation and advanced technology.

**Table 2:** Left part: Top-20 cities; right part: Bottom-20 cities. Cities in italics have residuals lower than 0.15 in the case of the top-20, and higher than -0.15 in the case of the bottom-20.

Prognos ranking	residual	kreisfreie city	Prognos ranking	residual	kreisfreie city
1	0.42	München	87	-0.21	Remscheid
2	1.05	Ingolstadt	88	-0.68	Delmenhorst
3	1.15	Wolfsburg	89	-0.18	Lübeck
4	0.68	Erlangen	90	-0.46	Halle
5	0.57	Stuttgart	91	-0.36	Suhl
6	0.46	Darmstadt	*92*	-0.15	Krefeld
7	0.71	Frankfurt aM	93	-0.51	Gera
8	0.65	Regensburg	94	-0.27	Hagen
9	0.21	Heidelberg	*95*	*-0*.*07*	*Wilhelmshaven*
10	0.50	Ulm	96	-0.33	Cottbus
11	0.26	Hamburg	97	-0.17	Schwerin
12	0.53	Düsseldorf	*98*	*-0*.*14*	*Neumünster*
13	0.63	Coburg	99	-0.26	Duisburg
*14*	*-0*.*27*	*Dresden*	100	-0.46	Oberhausen
15	0.28	Landshut	101	-0.17	Pirmasens
16	0.30	Würzburg	102	-0.40	Brandenburg ad H
17	0.30	Bamberg	103	-0.62	Herne
*18*	*-0*.*16*	*Jena*	104	-0.44	Dessau-Rosslau
*19*	*-0*.*01*	*Braunschweig*	*105*	*-0*.*12*	*Bremerhaven*
20	0.51	Bonn	106	-0.36	Gelsenkirchen

### Urban scaling and mono- versus polycentrality

With centrality we measure the extent to which population, or jobs, or GUP, are divided over all cities/towns within municipalities, or over municipalities within districts such as Kreise. We here focus on population-based centrality. A simple measure of centrality is c_1_, the ratio of the population of the largest city/town in the municipality to the total population of the municipality. In the municipalities of larger cities generally half of the total population or more lives in the central city. Thus, for typical *one-municipality* urban areas c_1_ will be mostly around or above 0.50. Such urban areas we call monocentric. This is the typical situation for the kreisfreie cities: they are *one-municipality as well as monocentric* urban areas.

An interesting question is whether in *multi-municipality* urban areas such as densely populated Kreise centrality influences urban scaling. In particular, we want to find out whether the more monocentric Kreise (particularly urban areas around one not-kreisfreie cities with generally 10 to 15 municipalities within the Kreis) perform better than the more polycentric Kreise. This may be a first indication that monocentrality and one-municipality work in the same direction. In a first approach to investigate this, we apply three simple population-related centrality measures (including the above discussed c_1_) and see if these simple measures show interesting effects. In the case of Kreise, c_1_ is defined as

$${\text{c}}_{1}=N(1)/\{\sum_{\left(z=1\right)}^{n}N(z)\}$$ (1)

where *N*(*z*) indicates the number of inhabitants of a municipality with rank *z* in population size, thus *z* = 1 is the largest municipality in a specific Kreis. As we see in Eq (1) we calculate c_1_ as the ratio of the population size of the largest city in the Kreis and the total population size of the entire Kreis.

Our second experimental centrality measure is somewhat more complicated and is based on the distribution of population over all municipalities within a Kreis. Thus, we need data on population size of all these municipalities. If the population distribution over municipalities within a Kreis is sharply peaked, then one municipality within the Kreis (mostly the municipality of the largest city/town) plays the leading role and we have a *monocentric* Kreis. With a flatter distribution we have a more *polycentric* Kreis. This situation suggests the use of the Zipf-distribution, a *size-rank* distribution, as a centrality measure. This size-ranking should theoretically follow a power law with exponent -1.0, but in reality this is not always the case [45], see for instance a recent meta-analysis of the size distribution of cities in a large number of countries [46]. So generally we can define the Zipf-distribution as

$$N(z)=1/z^{\alpha }$$ (2)

where *N(z)* is the population size of a city (or another entity) with rank *z* in the distribution function and α is the power law exponent of the distribution function with a value usually around -1.0. As discussed, we want to investigate to what extent the Zipf-distribution can be used as a measure of centrality. In [47] the Zipf-distribution of German cities is investigated on the federal level (entire country) as well as on the state level. There are no studies on the Zipf-distribution of municipalities within smaller regions such as Kreise, this paper present first results in this respect. There is limited amount of literature on, for instance, the Zipf-distribution of the centrality of words in a semantic network [48], but to our best knowledge no literature on the use of the Zipf-distribution to assess centrality of urban areas or other populated districts. Following the line of our above discussion, deviations from the Zipf-distribution power law exponent -1.0 can be used as an approximate centrality measure and a criterion for mono- or polycentricity. For instance, if the power law exponent is -1.5, the distribution is steeper and the Kreis is more monocentric. If the power-law exponent is less than -0.5 we have a flat distribution and the Kreis is more polycentric. We analyzed the Zipf-distribution of municipalities within 236 Kreise in West Germany which involves the population data of nearly 9,000 municipalities. As discussed earlier, we consider the use of the Zipf-distribution as a centrality measure as an experimental approach in order to find out if this approach produces significant results.

The measurement of the Zipf-distribution is not always straightforward, see S3 Appendix. Nevertheless we find an exponent with high significance (R^2^>0.90) for the vast majority of Kreise (see Fig A3.1 in S3 Appendix for examples) and on this basis we conclude that the calculation of the Zipf-distribution is useful in the analysis of centrality. Our third experimental simple centrality measure is the ratio of the population of the largest municipality to the population of the second largest municipality in a Kreis, denoted as c_2_, thus

$${\text{c}}_{2}=N(1)/N(2)$$ (3)

Also here we would like to find out whether this simple measure makes sense. We calculated our three centrality measures for all 236 Kreise. This enables us to find out how the values of these measures are distributed over the Kreise and to identify Kreise with high or low vales of these measures. Thus, for each of the centrality measures we calculated the scaling for those Kreise that are in the top-25% and those that are in the bottom-25% of the centrality distribution. For clarity, we note that this is not a comparison between kreisfreie cities and Kreise, as in Fig 2, but a comparison between two subsets of Kreise. As shown in S2 Fig, upper left panel, we find that the scaling of the Kreise within the top-25% and those within the bottom-25% of the Zipf-exponent values show similar exponents (1.17, 95%CI [1.16–1.18], R^2^ = 0.92; and 1.14 (95%CI [1.12–1.16], R^2^ = 0.90, respectively), but the Kreise in the bottom-25% underperform (i.e., their absolute GUP values are lower). The same is the case for centrality measure c_1_ (S2 Fig, upper right panel): exponent top-25% is 1.18 (95%CI [1.16–1.19], R^2^ = 0.90) and for the bottom-25% it is 1.16 (95%CI [1.15–1.18], R^2^ = 0.91), thus similar exponents but different GUP values, again the bottom-25% underperforms. We performed random sampling data tests [21, 49] and found that the difference in GUP between Kreise in the top-25% and the bottom-25% is small but significant for both the Zipf-exponent as well as for c_1_. In contrast with these two centrality measures, we find for our third centrality measure c_2_ (S2 Fig, lower panel): exponent top-25% is 1.14 (95%CI [1.13–1.16], R^2^ = 0.89) and for the bottom-25% it is 1.12 (95%CI [1.10–1.14], R^2^ = 0.89). Thus again similar exponents, but in this case no significant difference in GUP values between top-25% and bottom 25% is found.

If we look at the distribution of these three experimental centrality measures over the Kreise, we find that both the Zipf-exponent values as well as the c_1_ values make a sharper distinction between the high and the low values than in the case of c_2_, and this is also evident if we compare the top- and bottom 10% with the top- en bottom-25% (data available in osf.io/4ru96). For this reason, the observations with the Zipf-exponent and with c_1_ seem more meaningful. The results suggest that the Kreise with a higher degree of monocentrality perform better. These differences seem small at first glance when looking at the figures, but given the logarithmic scale, the difference in GUP is quite substantial (about 14% in GUP). Nevertheless, more empirical work is needed to gain further insight into the role of centrality, and in particular the supposed benefits of monocentrality.

### Generative versus distributive processes

**Fig 5:**
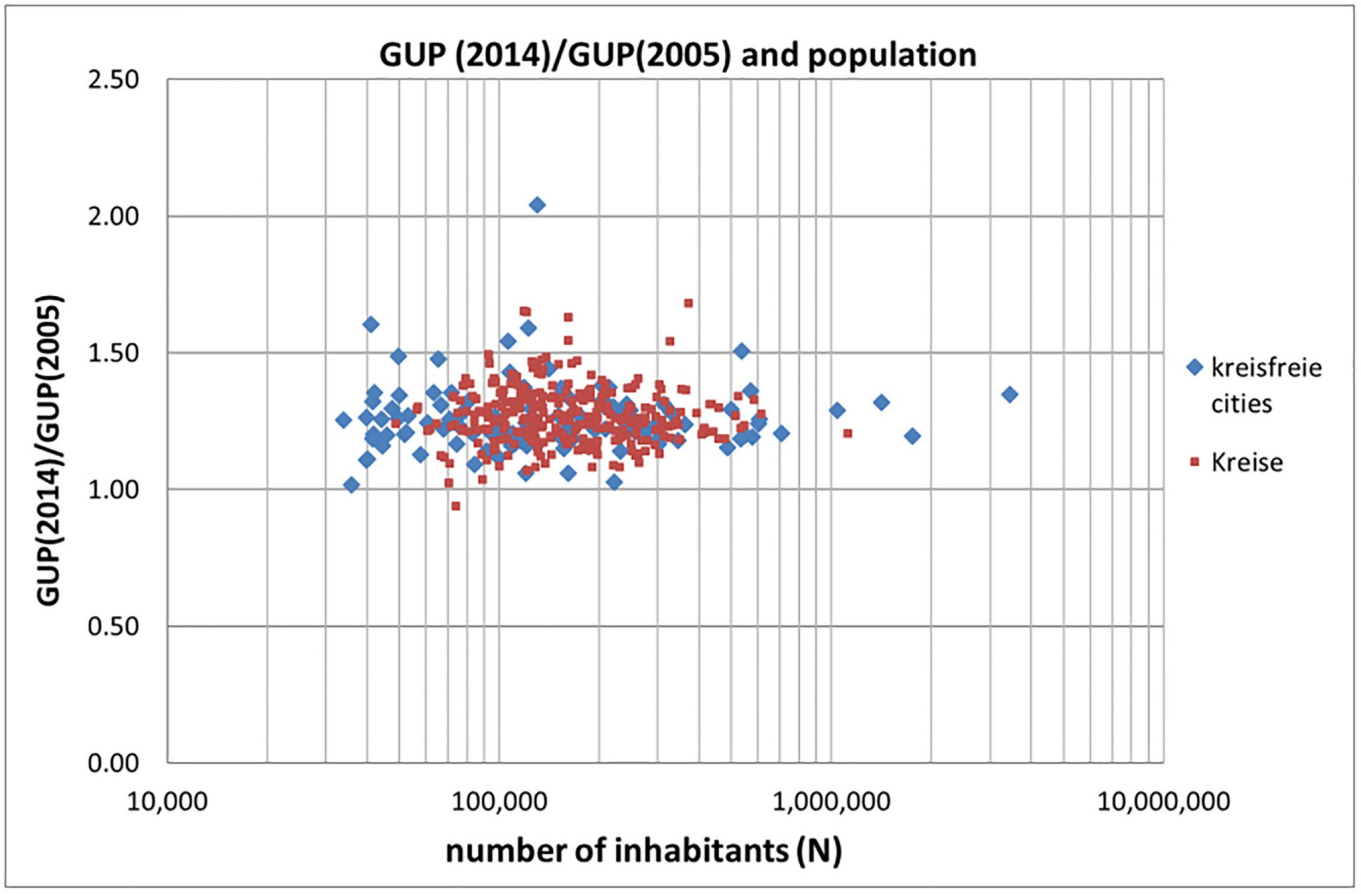
Increase of GUP of the kreisfreie cities in the period 2005–2014 as a function of population size.

One might ask whether the superlinear increase of GUP as a function of population size benefits the larger cities at the cost of smaller cities. In that case scaling would (also) be a result of *distributive* processes instead of *generative* processes (cities of all sizes benefit). To answer this question we calculate the increase of GUP over a period of, for instance 10 years. There must be a clear positive dependence on population size if scaling would involve distributive processes. Fig 5 shows that this is not the case. For both the kreisfreie cities as well as the Kreise no significant dependence of the ratio GUP(2014)/GUP(2005) on population size is found. We have similar results for all Danish municipalities, see S1 Appendix. Given the large amount of cities and Kreise and the broad range in population size in the generative versus distributive analysis, the time period involved (10 years) is sufficient.

Our results seem to follow Gibrat’s Law (population growth rate of a city does not depend on the population size of the city) but they are different because our results are about the *increase of GUP* as a function of *population size* and not about the increase of population as a function of population nor the increase of GUP as a function of GUP. This latter would indeed be a form of Gibrat’s Law [50–54]. But as discussed, that is not the case here. We conclude that urban scaling is related to generative processes.

## Conclusions and policy implications

### Research related to municipal reform

There is a vast amount of literature on municipal reform (also called ‘municipal amalgamation’ or ‘municipal mergers’) but to the best of our knowledge there is no work on municipal reform that take urban scaling, i.e., the disproportional increase of the gross urban product as a function of population, into account. Municipal reform literature focuses on the cost-side and hardly on the profit. Danish work [33, 55] focuses on the financially opportunistic behavior in terms of ‘last minute’ spending at the end of the budget year by the ‘old’ municipalities in the years before the Danish municipal reform in 2007 took place. Similar ‘free-riding’ spending behavior was found for municipal reform processes in Sweden [56, 57]. In this context it was concluded that municipal reform will only be successful if long-term benefits of municipal reform outweigh the short-term costs associated with them [58]. Also for the Finnish municipal reform around 2008 the free-riding behavior was studied [59] and the authors suggest financial constraints during the process of merging municipalities to mitigate this problem. In a further Danish study [60] it is found that an increasing municipal size through municipal reform initially has an adverse effect on fiscal outcomes, but there were improvements after four to five years. Particularly size effects persist over time. Their overall conclusion is that rescaling local government through municipal reform seems to improve economic steering capacities and fiscal outcomes.

In another Danish study [61] the authors conclude on the basis of survey data collected before and after the Danish municipality reform that when the size of municipalities increases, internal political efficacy drops. We think these findings are questionable. First, the survey data of the opinions of citizens concerning the municipal reform (in 2007) were collected practically immediately after the reform in 2008 (and partly even in the same year of the reform 2007). Municipal reform often evokes strong emotions of lost identity and feelings of resentment and it may take several years before citizens become aware of the benefits. Moreover, decrease of political efficacy conflicts with the fundaments of urban scaling: the disproportional increase of social, economic and cultural connections as a function of size. In a study on the municipal reform in New Zealand [62] no evidence was found that municipal reform did improve highway maintenance. Although their analysis is restricted to a just a very specific part of the efficiency in government operations, these authors used their findings to claim that there are no economies of scale. Also in a Swiss study [63] the authors conclude that there are no economies of scale in merged municipalities but also this analysis concerns only the cost side.

On the basis of the analysis of the expenditures of merged municipalities Finnish authors [64] conclude that the per capita expenditure increased more in the merged municipalities than in the comparison group of not-merged municipalities. Here we notice that urban scaling means a disproportionally increase of the gross urban product and thus it is understandable that the merged municipalities have more to spend. In an Israeli study the author finds evidence for efficiency gains arising from municipal reform in his country [65] and also that municipal reform resulted in lower levels of expenditures and has not seemed to decrease the quality of services. Although the empirical work is restricted to relatively small municipalities, this author suggests that as municipalities’ size increases the benefits arising from a municipal reform decrease. Apart from the fact that only the cost side and not the benefits are looked at, we also found no indications whatsoever in the literature that a clear distinction is made in types of municipal reform, particularly the merging of suburban (i.e., agglomeration) municipalities with the central city in a compact and densely connected urban area, versus the merging of more rural and less densely connected municipalities. In a German study [66] the fiscal consequences of both compulsory and (semi-) voluntary municipal mergers was investigated. These authors find substantial and immediate reductions in total expenditures, particularly after compulsory mergers.

In most earlier work on urban scaling the ‘cities’ are in fact larger agglomerations around central cities. In these studies the authors emphasize [2] that these agglomerations with its many municipalities are socio-economic units and therefore the defining feature of ‘cities’, this in contrast to governance-related definitions (such as municipalities) which are regarded as more arbitrary. We however argue in this paper that the political governance structure of urban agglomerations does have a great influence on the socio-economic strength of cities and their agglomerations. This urban governance structure often has very longstanding and deep historical, political and social grounds that are frequently the basis of emotional attitudes against the central city. Emotions are related to issues such as identity, supposed threats (lower income housing, higher taxes, the loss of green space and local facilities), and proximity of local authorities.

### Policy implications of our results

In this study we have investigated the scaling behavior (scaling exponent and GUP values) of all Danish municipalities (S1 Appendix), and particularly all German kreisfreie cities (major cities of which the surrounding urban area belongs to the municipality of the city) and all Kreise (areas around smaller cities consisting of separate municipalities). For the Netherlands (S2 Appendix) we analyzed the group of major cities including their urban agglomerations (consisting of separate municipalities). In the case of Denmark we analyzed the scaling of larger municipalities, municipalities within the Copenhagen agglomeration, and municipalities with high and low centrality. In all cases superlinear scaling of the gross urban/municipal product with population was found with scaling exponents between 1.14 and 1.24. Also in the case of Germany we find significant superlinear scaling of the gross urban product with population size with exponents up to 1.33. But the most interesting finding of our analysis is that urban areas with *one municipality* (one-governance, kreisfreie cities) perform better than urban areas with *fragmented governance* structures (more than one municipality, multi-governance). Furthermore, we find a strong relation between the measured residuals of the scaling equations and the socio-economic position of a cities as measured with a combination of different socio-economic indicators.

For the 21 major cities with their agglomerations in the Netherlands again significant superlinear scaling is measured with exponents up to 1.27. Our earlier observation that one-governance urban areas perform better than multi-governance urban areas is confirmed and this is in line with the findings for Germany in this study. Interestingly, it is again not the exponent of the scaling equation that makes the difference but the value of the GUP. Our conclusions are reinforced by the new finding that the more municipalities there are in an urban agglomeration of a certain population size, the larger the negative effect in terms of GUP as compared to a one-governance city with the same population size (Fig A2.5 in S2 Appendix). We again emphasize that our observations do not allow a strict identification of causality. However, our results show that a higher GUP occurs in significantly more cases when a specific modality of local government—namely a one-municipality urban area- is present than in cases where it is absent.

Undoubtedly, the independent municipalities within urban agglomerations will have socio-economic connections. But this does not mean that this multi-governance structure within these agglomerations has a strong cohesiveness and synergy resulting in an optimal social, economic and cultural coherence. Quite the contrary, the independent, autonomous municipalities within urban agglomerations each have their own political and social agenda. Even a medium-sized compact urban area may consist of eight autonomous municipalities with in total about 400,000 inhabitants (the case of Leiden). Every four years there are new municipal elections which may involve a complete change of political orientation. This often results in new policy making in which previous collaboration agreements and partnerships between the central city and all agglomeration municipalities may be revised or even eliminated thereby eroding the culture of mutual confidence. As a consequence, urban agglomerations may suffer considerably for many decades from the lack of vigor and perseverance in the realization of infrastructural, social, cultural and economic (particularly industrial business parks) facilities. The probability that a compact urban region will suffer from a less coherent urban governance is larger in the case of more municipalities.

Our observations in this study lead to challenging conclusions about the importance of a one-municipality instead of a multi-municipality governance in major urban areas. In a recent OECD report on urban governance the authors state that the effects of governance fragmentation in urban agglomerations are hardly discussed in the existing urban science literature although the influence of governance structures on urban socio-economic performance is generally thought to be pervasive [30]. This OECD work focusses on governmental fragmentation, its administrative complexity and coordination difficulties. The authors state that their report is a first attempt in the literature to empirically examine the relationship between urban governance structure and city productivity. The authors report that fragmented governance often results in problems related to coordination across the local governments. Thus fragmentation induces a high degree of coordination complexity which may hamper the enhancement of socio-economic strength in an urban agglomeration. In particular, urban agglomeration governance can obstruct necessary transport infrastructure investments and effective land use planning [67]. Furthermore, the additional bureaucracy associated with fragmented governance may also pose problems in the area of business and environmental regulation which also may impede socio-economic growth [68, 69]. In [70] it is concluded that the more closely the urban government and functional boundaries of an urban area correspond, the more likely it will be that effective local growth promotion policies develop. These findings are supported by recent research into the inhibition of prosperity by municipal borders [71]. The OECD authors [30] find that cities with fragmented urban governance structures tend to have lower levels of productivity. For a given population size, an urban area with twice the number of municipalities is associated with around six percent lower productivity. The authors even call this a ‘fragmentation penalty’. We agree with these authors that a full examination of the causes of lower productivity in urban areas with fragmented governance requires further studies on urban governance structures.

A more coherent governance of major cities and their agglomerations may create more effective social interactions which reinforce economic and cultural activities generating a substantial wealth benefit. Even in the case that only a part of the differences in performance between one- and multi-governance urban areas can be explained by incoherent governance, then still a substantial part of the expected benefits would generate a significant increase of wealth and disposable resources. For instance, in the case of the Netherlands (see S2 Appendix), if the benefit would be only 10% of the expected value, then we are still talking about a hundred million Euros per medium-sized city, which means thousands of jobs.

Inter-municipal collaboration is meant to improve the relations between central cities and their suburban municipalities, but to our knowledge there is hardly any study that shows how far the improvements would go if the urban area would change into a one-governance structure. This study provides strong indications for the benefits of a one-governance structure. These indications are in accordance with the findings of the above discussed OECD study on the role of urban governance in making cities more productive [30]. Increasing size of cities lead to greater professionalization, higher organizational specialization and increased administrative capacity. But above all, increasing size means a disproportional increase of socio-economic strength. Just like a recent study on urban areas as drivers of economic growth [72] our work underlines the importance of more effective governance in compact urban areas. The lack of coherent urban governance may severely hamper developments in national wealth. As discussed above, results of recent research on the geography of employment shows that the municipal boundaries may significantly limit welfare [71].

## Materials and methods

### Data sources

The data on gross urban product and population at municipality level were provided by Danmarks Statistik (Statistics Denmark) [35]. Surface areas of all Danish municipalities and the population size of the main city/town in the municipalities are retrieved from the Wikipedia websites of the municipalities [36]. We have randomly checked these data with data on the official website of the municipalities concerned. For a map of the Copenhagen agglomeration we refer to [73]. The data on gross urban product and population at the level of the German kreisfreie cities and Kreise and also data on population and surface area for all municipalities in the Kreise were provided by the Statistisches Bundesamt (German Federal Bureau of Statistics) [35]. Surface areas of all German kreisfreie cities and Kreise are retrieved from the Wikipedia websites of the cities and Kreise [74]. The data on the gross urban product of municipalities in The Netherlands are obtained from the national information system on employment LISA, and the data on the population of municipalities from Statline, the data system of the Netherlands Central Bureau of Statistics (CBS) [37].

### Calculation of the residuals

We calculated the residuals of the power-law scaling of the gross urban product with population for the analysis of the real performance as compared to the expected value. The mathematical procedure is as follows. A power-law relation between for instance the gross urban product (*G*) and population (*P*) can be written as:

$$G(P)=aP^{\beta }$$

The values for the coefficient *a* the power-law exponent ẞ follow from the empirically determined scaling equation.

Denoting the observed value of the gross urban product for each specific city (municipality) with *G*_i_, we calculate the residuals *ξ*_i_ of the scaling distribution of each city as follows [2, 21]:

$$\xi_{i}=ln[G_{i}/G(P)]=ln[G_{i}/aP^{\beta }]$$

The residuals are also used to test the heteroscedasticity of the data, we refer to our earlier paper [21].

## Supporting information

S1 Fig Scaling of all kreisfreie cities, comparison by region. (TIF)

S2 Fig Scaling of the Kreise in relation to three experimental centrality measures. Upper left panel: top- and bottom-25% of the Zipf exponent values. Upper right panel: top- and bottom-25% of the c_1_ values. Lower panel: top- and bottom-25% of the c_2_ values.
(TIF)

S1 Appendix Scaling of the Danish municipalities. (DOCX)

S2 Appendix Scaling of major cities and their agglomerations in the Netherlands. (DOCX)

S3 Appendix Zipf-distribution. (DOCX)
