# Supplementary figures and images for: Urban scaling, geography, centrality: Relation with local government structures
Source: PLoS One. 2020 Sep 4;15(9):e0238418. doi: 10.1371/journal.pone.0238418 (PMC7473566; doi:10.1371/journal.pone.0238418)

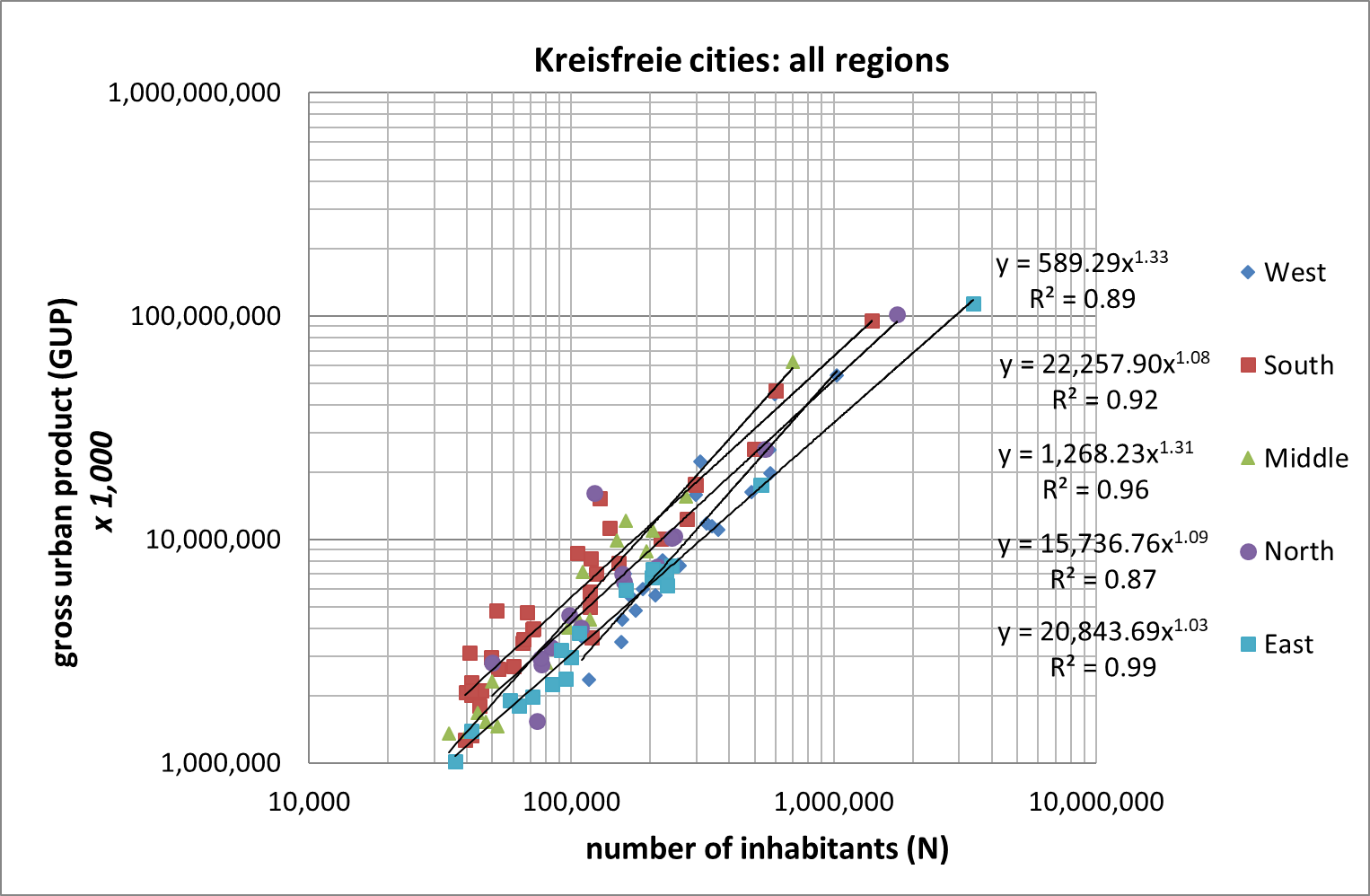

Supplement: S1 Fig — (TIF) [file pone.0238418.s001.tif]

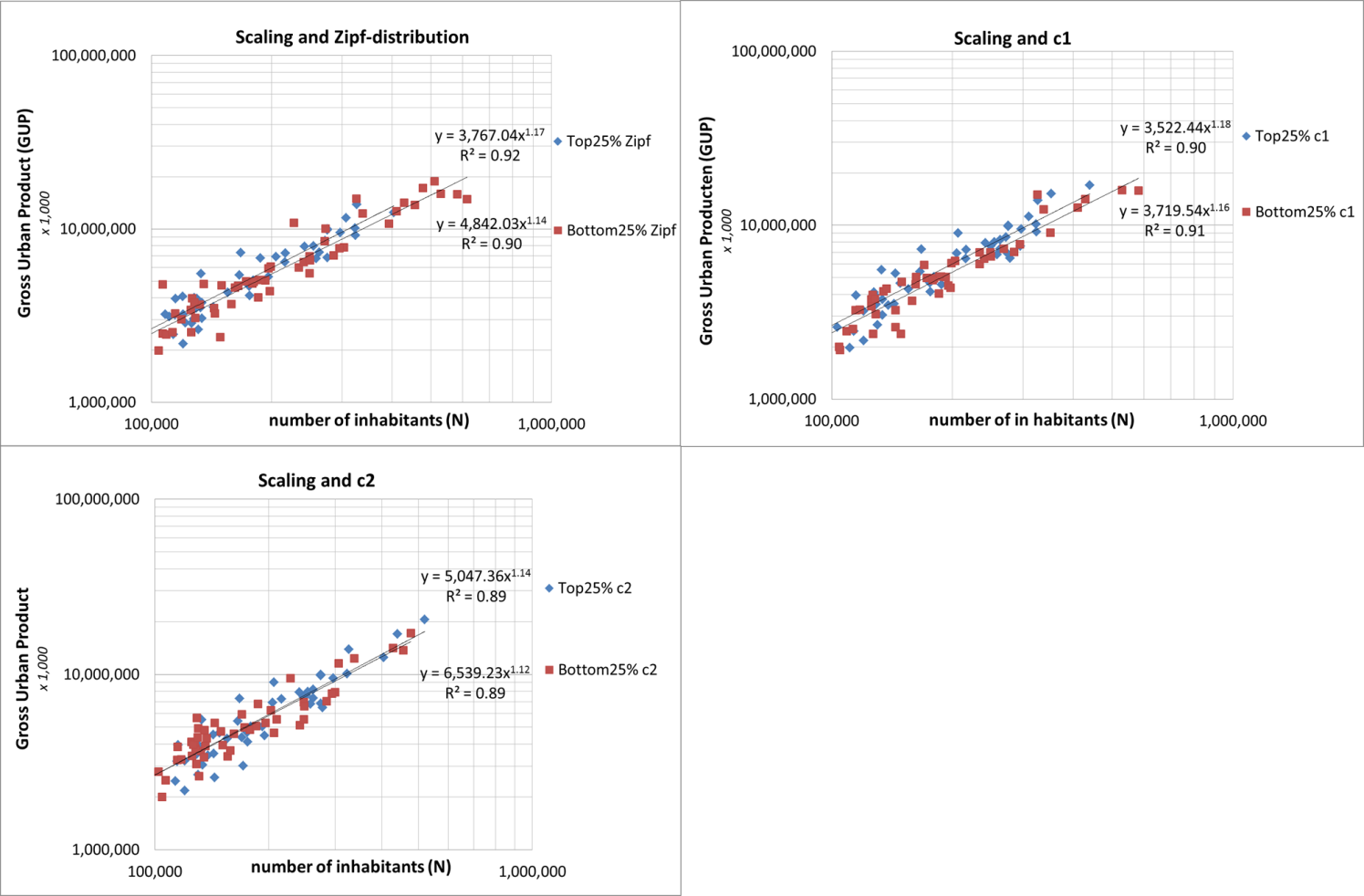

Supplement: S2 Fig — Upper left panel: top- and bottom-25% of the Zipf exponent values. Upper right panel: top- and bottom-25% of the c1 values. Lower panel: top- and bottom-25% of the c2 values. (TIF) [file pone.0238418.s002.tif]
