# Supplementary material for: Urban scaling, geography, centrality: Relation with local government structures
Source: PLoS One. 2020 Sep 4;15(9):e0238418. doi: 10.1371/journal.pone.0238418 (PMC7473566; doi:10.1371/journal.pone.0238418)
Supplement: S1 Appendix — (DOCX) [file pone.0238418.s003.docx]

S1 File Appendix 1: Scaling of the Danish Municipalities

We first analyzed the scaling of the gross urban (municipal) product as a function of population for the entire set of 98 municipalities and show the results in Fig A1.1, left panel. We find that the scaling exponent is 1.16 (95% level of confidence and R^2^ for the scaling exponents in this section are given in Fig A1.2). In some cases the deviation of an individual municipality from the regression line can be remarkably large. In the measurement of urban scaling the effect of such ‘outliers’ is an important issue. Indeed, as the figure suggests, the measured exponent is sensitive to outliers. There are several statistical tests to find out whether a data point can be considered as an outlier, but in most cases typical outliers are immediately visible in the scaling figure. A striking example is Billund (indicated with a circle in Fig A1.1, left panel), a small municipality of about 26,000 inhabitants but with a GUP four to five times higher than other municipalities of similar size. The reason for this exceptionally high GUP is obvious: Billund is the home town of Legoland, the largest tourist attraction in Denmark with two million visitors per year. Furthermore, Billund International Airport is the second largest airport in Denmark. Billund has by far the largest relative increase of GUP of all Danish municipalities in the last ten years. If we remove Billund as an exceptional outlier the exponent is 1.18. Other outliers are two municipalities within the Copenhagen agglomeration, Ballerup and Glostrup. Ballerup has the highest concentration of companies and jobs after Copenhagen. Glostrup has twice as many employees as compared with municipalities of similar size because several major international companies are located in Glostrup. Removing all three outliers results in a scaling exponent of 1.20. Removing all (28) municipalities of the Copenhagen agglomeration yields a scaling exponent 1.14. However, these differences in scaling exponent fall largely within the error margins.

Next, we investigate the scaling behavior for specific *subsets* within the total set of all municipalities. To the best of our knowledge this has been done so far only in the study of Polish cities where, just like in our work, the city is defined as a municipality [1]. The crucial question here is: do the urban scaling exponents, apart from the above mentioned effect of outliers, also depend on specific characteristics of the set of municipalities for which the scaling is measured? To investigate this, urban scaling of the following subsets is measured. Subset 1: municipalities with a population over 50,000; this means a subset of the larger municipalities; subset 2: municipalities within the Copenhagen agglomeration; this means a subset of municipalities that are very close together.

The next two subsets are based on centrality. We apply the following measure of centrality: the ratio of the population of the main city or town in the municipality to the total population of the municipality. In municipalities around larger cities at least half of the total population lives in the central city and the centrality will be above 0.50, a typical ‘monocentric’ municipality. The municipalities with centrality >0.50 form subset 3. In rural regions, the main towns of municipalities are often small with less than 20,000 inhabitants and the remaining inhabitants are divided over a larger number of smaller towns within the municipality. As a consequence, the centrality of rural regions municipalities is smaller than 0.50: ‘polycentric’. These municipalities with centrality <0.50 form subset 4.

The result for the first subset, all municipalities (n=37) with a population over 50,000 is presented in Fig A1.1, left panel. We find that the scaling exponent is 1.14 and this is statistically not different from the scaling of all municipalities. If we remove the municipalities in the Copenhagen agglomeration with more than 50,000 inhabitants the scaling exponent is 1.12, and this is within the error margin. Also in the same figure we show the scaling of the 28 municipalities in the Copenhagen agglomeration [2]. We find a scaling exponent 1.24. This means that also *within a compact urban area* the autonomous municipalities exhibit scaling behavior. We notice the outlier positions of two earlier mentioned Copenhagen suburbs, Glostrup and Ballerup. Because these agglomeration municipalities are located at the lower left side of the regression line they ‘lift’ as it were the regression line which causes a lower exponent if they are included, and a slightly higher exponent if they are excluded (1.28).

We see that in the Copenhagen agglomeration the power-law exponent is higher than in the case of all municipalities and in the case of the municipalities above 50,000 inhabitants. This raises an interesting question: is this higher exponent of urban scaling caused by the fact that the 28 municipalities in the Copenhagen agglomeration are very close together, and thus the whole subset can be seen as a strongly interconnected network of municipalities? If we leave out Copenhagen municipality, the scaling exponent is even higher, 1.30 (just within the error margin). It may suggest that Copenhagen municipality performs relatively less within the entire agglomeration. This raises the further question whether this finding has consequences for the political strategy to abandon municipal reform of the Copenhagen agglomeration. Because we have in Denmark only one large urban agglomeration (Copenhagen) we further investigate urban scaling within several major urban agglomerations in the Netherlands, see section “Scaling within Urban Agglomerations” in S2 File Appendix 2.

Next to scaling exponents we also notice that the Copenhagen agglomeration regression line is above those of the two other sets. In other words, the GUP values are higher. This suggests that the municipalities in the Copenhagen agglomeration overperform the municipalities in the other sets. And this can be expected as the Copenhagen agglomeration is the economic center of the country.

**
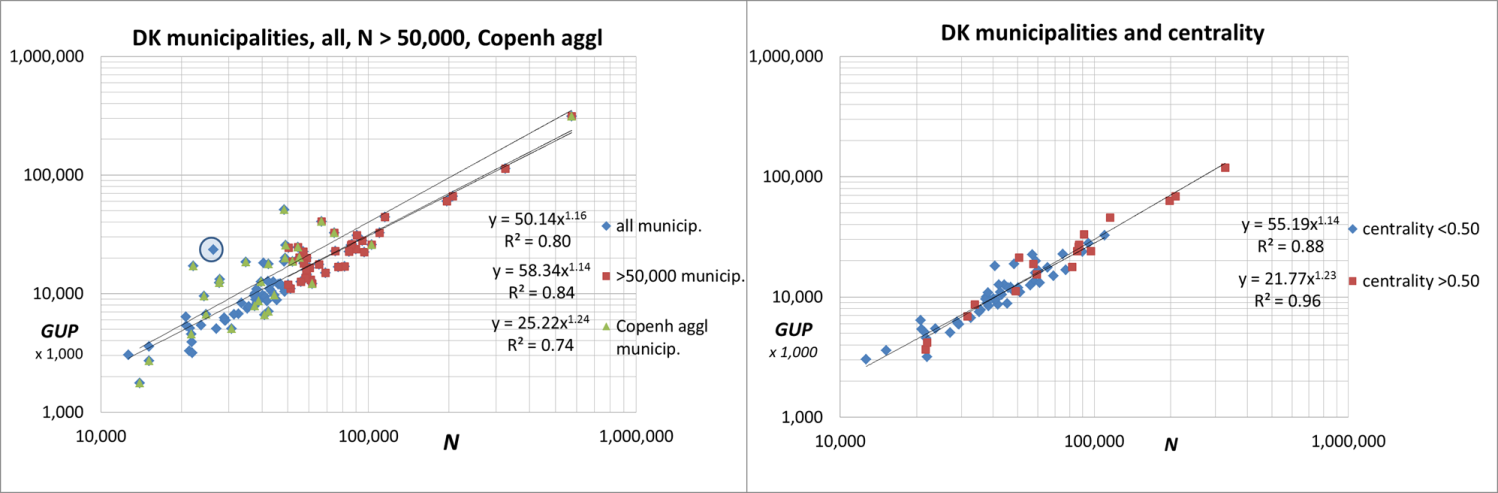
**

**Fig A1.1 Left: scaling of GUP for all Danish municipalities (blue diamonds), municipalities above 50,000 inhabitants (red squares), and municipalities within the Copenhagen agglomeration (light green triangles). Right: scaling GUP for all Danish municipalities with centrality <0.50 and not in the Copenhagen agglomeration (blue diamonds) and centrality >0.50 and not in the Copenhagen agglomeration (red squares) (GUP is in units of 1,000 DDK, data average 2013-2015). N is the number of inhabitants.**

The third and fourth subsets relate to centrality. The results are shown in Fig A1.1, right panel. For the municipalities with centrality <0.50 and not within the Copenhagen agglomeration (n=49, half of all Danish municipalities, we excluded Billund) we find a scaling exponent 1.14, not significantly different from the scaling exponent of all Danish municipalities. For the municipalities (again Copenhagen agglomeration excluded, in total 18 municipalities) with centrality >0.50 -most of the larger municipalities- we find a scaling exponent of 1.23, somewhat higher than in the case of municipalities with centrality <0.50. We give an overview of our findings in Fig A1.2.


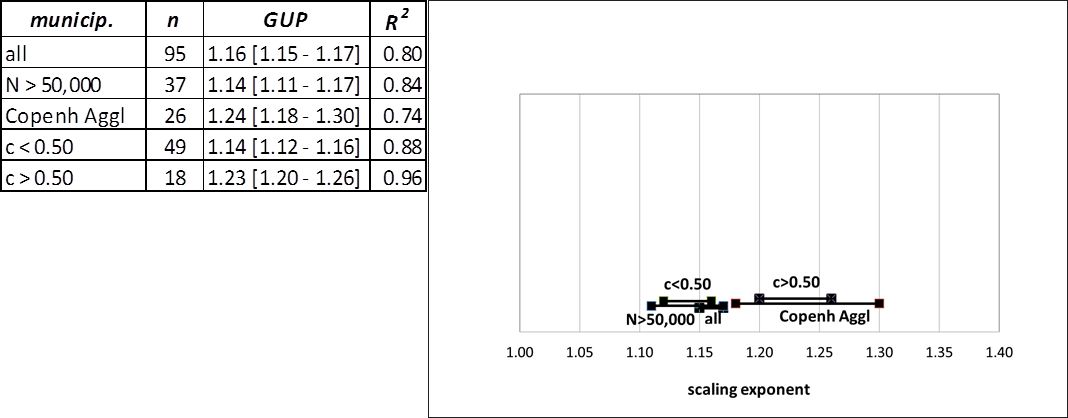


**Fig A1.2 Summary of GUP scaling exponents for Danish municipalities (square brackets indicate 95% CIs on scaling exponents); *n* is the number of municipalities.**

Just like in the case of Germany (see main text) we also investigated for the Danish municipalities whether the non-linear increase of GUP as a function of population size benefits the larger cities at the cost of smaller cities. Thus, scaling would be a result of *distributive* processes instead of *generative* processes. These latter imply that cities of all sizes benefit. Like in the German case we calculated the increase of GUP over a period of 10 years.


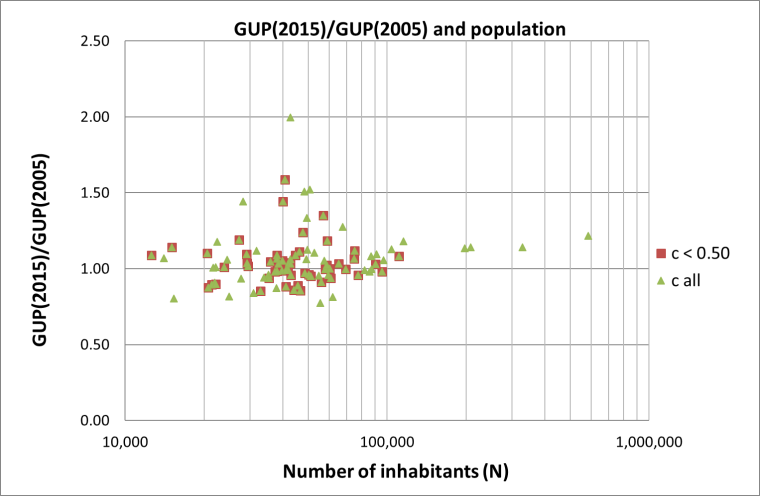


**Fig A1.3 Increase of GUP for all Danish municipalities and for those with centrality <0.50 in the period 2005-2015 as a function of population size.**

Fig A1.3 shows that no significant dependence of the ratio GUP(2015)/GUP(2005) on population size is found, and this is also the case for the subset of the Danish municipalities with centrality <0.50 which are predominantly the more rural municipalities. Again we conclude that urban scaling is a generative process.

References

1. Cebrat C, Sobczyński M (2016). Scaling Laws in City Growth: Setting Limitations with Self-Organizing Maps. *PLoS ONE* 11(12): e0168753.

2. Wikipedia, data on the Copenhagen Agglomeration available from: [https://en.wikipedia.org/wiki/Capital_Area_of_Denmark#Municipalities_of_Area_Hovedstaden](https://en.wikipedia.org/wiki/Capital_Region_of_Denmark#Municipalities_of_Region_Hovedstaden) and <https://en.wikipedia.org/wiki/Urban_area_of_Copenhagen>.
