# Supplementary material for: Urban scaling, geography, centrality: Relation with local government structures
Source: PLoS One. 2020 Sep 4;15(9):e0238418. doi: 10.1371/journal.pone.0238418 (PMC7473566; doi:10.1371/journal.pone.0238418)
Supplement: S2 Appendix — (DOCX) [file pone.0238418.s004.docx]

S2 File Appendix 2: Scaling of major cities and their agglomerations in the Netherlands

For major cities in the Netherlands the Central Bureau of Statistics (CBS) defines two types of agglomerations. First, the *urban agglomeration* consisting of the central city (which is a municipality) and the immediately connected suburban cities that are separate municipalities. Second, the *urban area* in which in addition to the urban agglomeration all other suburban cities (again separate municipalities) that are closely socio-economically connected to the central city are included. The largest urban area, Amsterdam, counts 1.7 million inhabitants. We made a scaling analysis for *three* urban modalities: (1) the major cities ‘alone’, i.e., as a municipality (in total 21); (2) their urban agglomerations (in total the 21 central cities and 40 suburban cities); and (3) their urban areas (in total the 21 central cities, the 40 suburban cities in the agglomerations, and in addition 89 suburban cities to complete the urban areas).

In Fig A2.1, left panel, we present the scaling of GUP for these 21 major cities and for their agglomerations and urban areas. We see that the major cities scale with the following exponents: 1.20 (95%CI [1.17-1.23], R^2^=0.94) for cities as a municipality (*CC*); 1.16 (95%CI [1.10-1.22], R^2^=0.92) for the urban agglomerations of these cities (*Aggl*), and 1.17 (95%CI [1.14-1.20], R^2^=0.94) for the urban areas of these cities (in these two latter cases: multi-governance structure). Our measurement suggest a slight decrease of the exponent from central cities as municipalities to urban agglomerations and urban areas, in agreement with our earlier analysis (period 2010-2012, now 2014-2016) [1]. But given the calculated error limits the significance of this decrease is low. It is not a difference in scaling exponents that is important in this measurement. What matters is the difference in GUP values.

The remarkable phenomenon discussed in [1] is confirmed: the value of the gross urban product for both the urban agglomerations and the urban areas is lower than for the central cities as municipalities. Thus, although both types of multi-governance urban area modalities scale with population, they underperform as compared to cities defined as municipalities. In other words, an urban area consisting of one municipality (one-governance) performs substantially better as compared with an urban agglomeration with the same number of inhabitants but consisting of several municipalities. And this difference is large in terms of GUP. For instance, the expected value for *CC* (one-governance) is in the case of 200,000 inhabitants around 20% higher than the expected value for *Aggl* (multi-governance). This gives a first indication of the profit that can be made by the municipal merging of a central city with its directly connected suburban municipalities in the agglomeration. Even if only a small part (say 10%) of these expectations is fulfilled, we are still talking about an amount of 100 million Euros per medium-sized city mainly in the form of thousands of jobs. An important further indication of the benefits of municipal merging can be found by analyzing the relation between the urban agglomeration residuals and the number of municipalities in the agglomeration. We will discuss this in section “Performance of urban agglomerations and number of municipalities”.

In Fig A2.1, right panel, we show the scaling of GUP for all the municipalities in the urban areas of the 21 major cities, in total 150, which is nearly half of all municipalities in the Netherlands. The most likely explanation of the high scaling exponent 1.27 (95%CI [1.26-1.28], R^2^=0.89) is that particularly the smaller suburbs are typical residential municipalities and consequently they have a relatively low GUP. This pulls down the regression line resulting in a higher exponent. We see several outliers, i.e., municipalities with an exceptionally high residual. We give two examples, see circles in Fig A2.1, right panel. The one at the lower end of the population scale is Zoeterwoude, a small municipality (population about 8,000) in the Leiden agglomeration. This outlier position is mainly due to the presence of the large Heineken beer factory. The outlier at the higher end of the population scale is Haarlemmermeer, a larger municipality (population about 150,000) in the Amsterdam urban area. Also this exceptional position is very well understandable, Amsterdam International Airport Schiphol, the fourth largest airport in Europe, is located in Haarlemmermeer. If Zoeterwoude is removed from the analysis, the scaling exponent is 1.28. Removal of Haarlemmermeer gives a scaling exponent of 1.25.


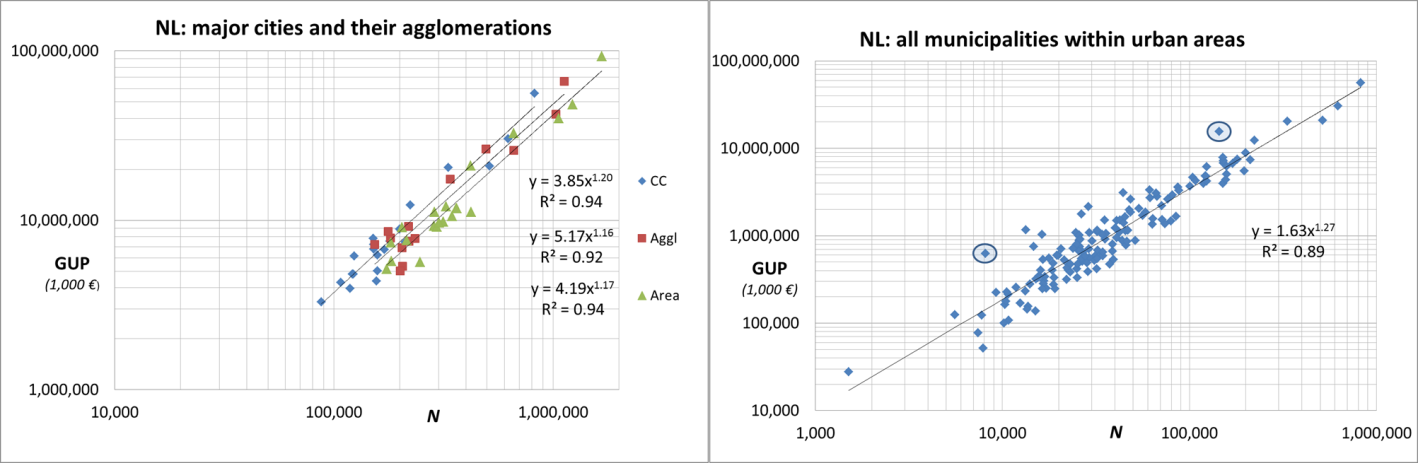


**Fig A2.1 Left: scaling of the 21 major cities with GUP. The central cities are indicated with blue diamonds (CC), their urban agglomerations with red squares (Aggl) and their urban areas with light green triangles (Area). Right: scaling of GUP for all 150 municipalities in the urban area of the 21 major cities. N is the number of inhabitants. Circles indicate examples of outliers, see discussion in the text.**

Scaling within Urban Agglomerations

We found that scaling of the municipalities within the Copenhagen agglomeration (see S1 File Appendix 1) follows a power-law exponent 1.24 (95%CI [1.18-1.30], R^2^=0.74). Given that the Netherlands has more larger urban agglomerations with suburban municipalities around the central city, we further analyzed the within-agglomeration scaling for the four largest urban areas in the Netherlands: Amsterdam (population 1,728,000), Rotterdam (1,256,000), The Hague (1,096,000) and Utrecht (682,000). The results of the scaling analysis are presented in Fig A2.2. We find that the scaling exponents of these urban areas are within a range between 1.10 (95%CI [0.98-1.21], R^2^=0.88) (The Hague) to 1.17 (95%CI [1.12-1.23], R^2^=0.92) (Rotterdam). The scaling exponents are somewhat lower as compared with the Copenhagen agglomeration, but nevertheless we conclude that scaling within urban agglomerations does not behave differently from urban scaling of, for instance, all cities (municipalities) or all urban agglomerations as a whole in a country. Scaling is apparently a fractal phenomenon.


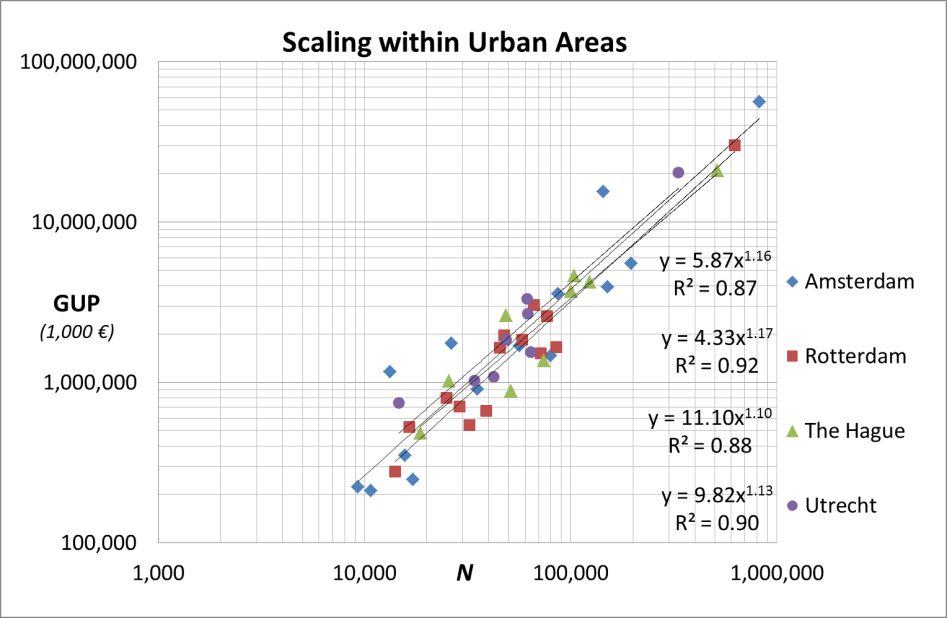


**Fig A2.2 Scaling of GUP for all municipalities within the urban areas of Amsterdam, Rotterdam, The Hague, and Utrecht. N is the number of inhabitants.**

Urban Agglomeration Related Residuals

South-Holland (PZH) is the most populated province of the Netherlands with 3,6 million inhabitants and 52 municipalities. In Fig A2.3 the scaling of GUP with population for all PZH municipalities is shown. The scaling exponent is 1.11 (95%CI [1.09-1.12], R^2^=0.89).


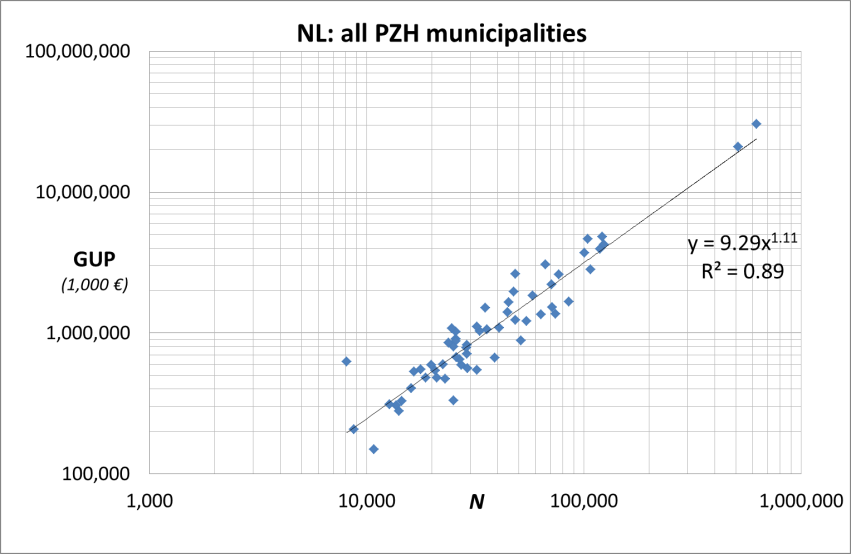


**Fig A2.3 Scaling of GUP for all PZH municipalities**

On the basis of the scaling equation in Fig A2.3 we calculated the residuals for all PZH municipalities. The results are shown in Fig A2.4. We see that of the four major cities in PZH, Rotterdam and Leiden have relatively large positive residuals, whereas the residuals for The Hague and Dordrecht are around zero. High positive residuals are found for Rijswijk (The Hague agglomeration) and Sliedrecht (Dordrecht agglomeration).

We now focus our analysis further on the above mentioned four major urban agglomerations in PZH (Rotterdam, The Hague, Leiden, Dordrecht) and calculate a set of specific residuals, see Table A2.1. The explanation of the table is as follows. In the first column the municipalities within the urban agglomerations are given. Directly below the central city we find the municipalities of the urban agglomeration (for instance in the case of Rotterdam these are the municipalities up to and including Krimpen aan den


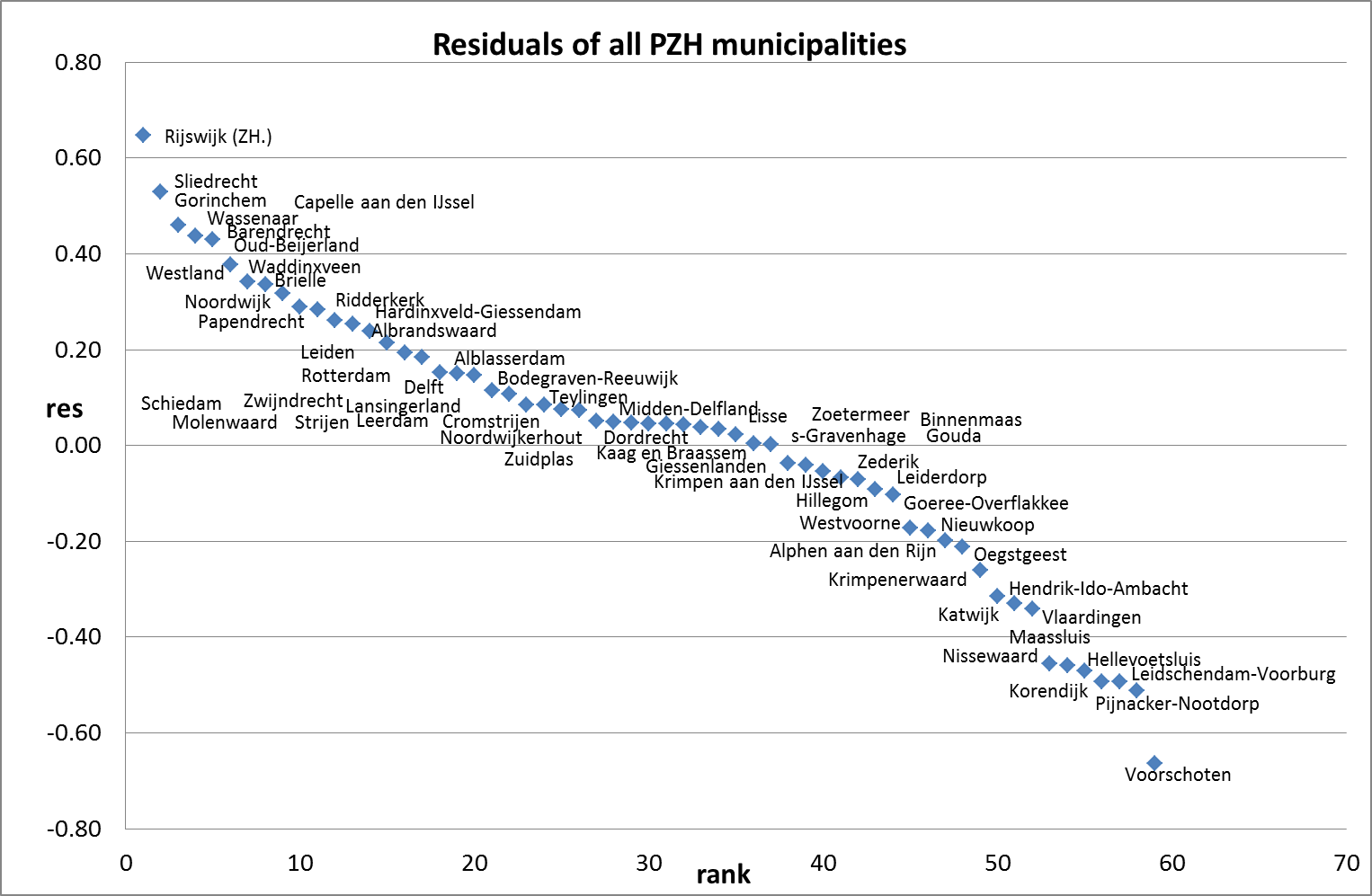


**Fig A2.4 Residuals of all PZH municipalities.**

Ijssel). The column *resAll* gives the values of the residuals of the four cities and their agglomeration municipalities in relation to the scaling of all 150 urban agglomeration municipalities in the Netherlands (Fig A2.1, right panel). In the next column we find *resCC*, the residuals in relation to the scaling of the 21 major cities in the Netherlands, i.e., the residuals calculated with the scaling equation of the CC-regression line in Fig A2.1, left panel. It is a measure of *over- or underperformance* of municipalities in a national context. We see that with the exception of Leiden, the other three cities (municipalities) Rotterdam, The Hague and Dordrecht substantially underperform in this national context. In comparison, the *resCC* values for other major cities in the Netherlands are for instance Amsterdam 0.18, Eindhoven 0.22 and Utrecht 0.25. The correlation between *resAll* and *resCC* is high (R^2^=0.97).

Residual *resUAg* is calculated with the scaling equation of the Aggl-regression line in Fig A2.1 (left panel). It is a measure of over- or underperformance of urban agglomerations (central city with the immediately connected suburban cities that are separate municipalities), again in a national context. The results are quite remarkable. The Rotterdam agglomeration does not improve as compared to the Rotterdam *resCC*, which means that currently the suburban municipalities are not in a position to reinforce the performance of the Rotterdam urban agglomeration. We observe a similar situation for Leiden. The opposite is found for the Hague and Dordrecht. The next residual is *resUAgCC*, in our opinion the most interesting measure. If an agglomeration would be a one-governance (i.e., one municipality) urban area (like the kreisfreie cities in Germany), the *expected position* should be on the CC regression line. Thus *resUAgCC* indicates the difference in gross urban product between what the agglomeration would ‘earn’ if it was a one-governance city, and what it actually earns now, in a fragmented, multi-governance urban agglomeration. In all four cases it is clear that the multi-governance structure does not succeed in attaining the expected one-governance level of the gross urban product. Calculations for the wider urban agglomerations, the urban areas (the Area regression line in Fig A2.1 left panel) reveal similar findings.

**Table A2.1. Residuals for the PZH major cities and their urban agglomerations.**

|  | ***resAll*** | ***resCC*** | ***resUAg*** | ***resUAgCC*** |
| --- | --- | --- | --- | --- |
| **Rotterdam** | -0.17 | ***-0.11*** | ***-0.17*** | ***-0.38*** |
| Schiedam | 0.06 | ***-0.05*** |  |  |
| Nissewaard | -0.52 | ***-0.62*** |  |  |
| Vlaardingen | -0.38 | ***-0.50*** |  |  |
| Capelle aan den IJssel | 0.41 | ***0.28*** |  |  |
| Barendrecht | 0.39 | ***0.24*** |  |  |
| Maassluis | -0.39 | ***-0.57*** |  |  |
| Krimpen aan den IJssel | 0.01 | ***-0.18*** |  |  |
|  |  |  |  |  |
| **Den Haag** | -0.29 | ***-0.25*** | ***-0.15*** | ***-0.34*** |
| Leidschendam-Voorburg | -0.54 | ***-0.65*** |  |  |
| Rijswijk (ZH.) | 0.66 | ***0.51*** |  |  |
| Wassenaar | 0.53 | ***0.32*** |  |  |
|  |  |  |  |  |
| **Leiden** | 0.09 | ***0.01*** | ***-0.11*** | ***-0.25*** |
| Leiderdorp | 0.02 | ***-0.18*** |  |  |
| Voorschoten | -0.57 | ***-0.77*** |  |  |
| Oegstgeest | -0.10 | ***-0.31*** |  |  |
| Zoeterwoude | 1.51 | ***1.22*** |  |  |
|  |  |  |  |  |
| **Dordrecht** | -0.08 | ***-0.16*** | ***-0.10*** | ***-0.25*** |
| Zwijndrecht | 0.14 | ***-0.02*** |  |  |
| Papendrecht | 0.32 | ***0.14*** |  |  |
| Sliedrecht | 0.63 | ***0.42*** |  |  |

We conclude this section with a remark about population densities. Are multi-governance urban areas (agglomerations) less densely populated than one-governance urban areas? If so, it could be an explanation why most of the urban agglomerations in the Netherlands are characterized by underperformance. But this is not the case. A detailed analysis in our earlier study [1] showed that population density does not relate to the measured residuals. In other words, over- and underperformance of cities as compared to the expected GUP values cannot be attributed to population density. The Rotterdam agglomeration has 8 municipalities, and the overall density is 2,680 inhabitants/km^2^. Compare this with two German harbor cities, Hamburg and Bremen, both are kreisfrei, i.e., central city plus agglomeration is one municipality. The densities for these *one-governance* urban areas are for Hamburg 2,304 inhabitants/km^2^ and for Bremen 1,682 inhabitants/km^2^, well below the *multi-governance* Rotterdam agglomeration. The densities of the multi-governance The Hague agglomeration (4 municipalities) and the multi-governance Leiden agglomeration (5 municipalities) are 3,656 and 2,775 inhabitants/km^2^, considerably higher than the population densities of Hamburg and Bremen or of Cologne and Düsseldorf with 2,542 and 2,744 inhabitants/km^2^, respectively. Again, we conclude that population densities are most probably not a main factor in the difference of GUP between one-governance and multi-governance urban areas.

Residual Analysis and Comparison with Socio-economic Data

As discussed in context of the scaling of German cities (see main text), analysis of the residuals may reveal characteristics of individual cities in terms of success or failure relative to other cities. Although different from the German research on the socio-economic perspectives of cities, also in the Netherlands similar research takes place, particularly a socio-economic assessment of the 50 largest cities published annually in the Atlas voor Gemeenten (AvG, Atlas for Municipalities). These assessments are based on a set of quantitative and qualitative indicators [2]. Of these 50 cities, 32 are within the urban agglomerations/areas of the 21 major cities (either as the central city, or as a major suburb). For these 32 cities we compared their residuals (*resCC*) with the ranking score in de socio-economic index of the AvG 2017. To avoid the influence of the individual residuals as well as individual AvG scores, we calculated average values for blocks of five cities according to the ranking of the residuals, see Table A2.2. For instance, in the second column 0.38 is the average residual of the top-5 cities (in terms of the residuals), and 18.0 is their average AvG score, and so on for the next blocks of five cities.

**Table A2.2. Comparison of residuals with scores in the AvG socio-economic review.**

|  | ***resCC*** | **av res** | **score** | **av score** |
| --- | --- | --- | --- | --- |
| Haarlemmermeer | 0.98 |  | 24 |  |
| Utrecht | 0.25 |  | 22 |  |
| Den Bosch | 0.24 |  | 19 |  |
| Zwolle | 0.23 |  | 17 |  |
| Eindhoven | 0.22 | 0.38 | 8 | 18.0 |
| Velsen | 0.18 |  | 12 |  |
| Amsterdam | 0.18 |  | 13 |  |
| Arnhem | 0.14 |  | -11 |  |
| Amstelveen | 0.12 |  | 23 |  |
| Amersfoort | 0.08 | 0.14 | 21 | 11.6 |
| Leeuwarden | 0.04 |  | -3 |  |
| Groningen | 0.03 |  | 2 |  |
| Heerlen | 0.02 |  | -21 |  |
| Leiden | 0.01 |  | 15 |  |
| Maastricht | 0.00 | 0.02 | -9 | -3.2 |
| Hengelo | 0.00 |  | -6 |  |
| Breda | -0.02 |  | 20 |  |
| Delft | -0.03 |  | 0 |  |
| Apeldoorn | -0.05 |  | 9 |  |
| Schiedam | -0.05 | -0.03 | -15 | 1.6 |
| Nijmegen | -0.06 |  | -8 |  |
| Rotterdam | -0.11 |  | -17 |  |
| Zoetermeer | -0.14 |  | 3 |  |
| Dordrecht | -0.16 |  | -19 |  |
| Tilburg | -0.22 | -0.14 | 7 | -6.8 |
| Den Haag | -0.25 |  | -16 |  |
| Enschede | -0.26 |  | -24 |  |
| Haarlem | -0.39 |  | 14 |  |
| Almere | -0.43 |  | -12 |  |
| Vlaardingen | -0.50 |  | -20 |  |
| Leidschendam-Voorburg | -0.65 |  | 4 |  |
| Purmerend | -0.67 | -0.45 | -1 | -7.9 |

We find a correlation of R^2^=0.79. This is a simple test but it confirms the findings in the case of Germany: there is a significant correlation between the measured residuals based on the GUP scaling and socio-economic indicators from other sources.

Performance of urban agglomerations and number of municipalities

As discussed earlier, the *resCC* residuals are calculated with the scaling equation of the CC-regression (Fig A2.1, left panel) and they provide a measure of *over- or underperformance of cities* (as municipalities) *in a national context*. If the *agglomeration of a central city* would be a one-governance city, the *expected position* should be on the CC regression line. Its real position is, as we discussed in the foregoing section, given by *resUAgCC*. The difference *∆res*=(*resAgCC*–*resCC*) is a measure of under- or over performance of an urban agglomeration relative to the performance of the central city of the agglomeration.

We now look at the relationship between *∆res* and the number of municipalities in the urban agglomeration for the 21 major cities in the Netherlands. Five cities do not have an agglomeration, for instance because the former agglomeration municipalities have been merged with the central city in recent times. In those cases *∆res*=0 and we set the number of municipalities in the urban agglomeration at 1. One of the 21 urban agglomerations is in fact a double city structure which makes it an exception compared to all other urban agglomerations. The result without the exceptional case is shown in Fig A2.5.


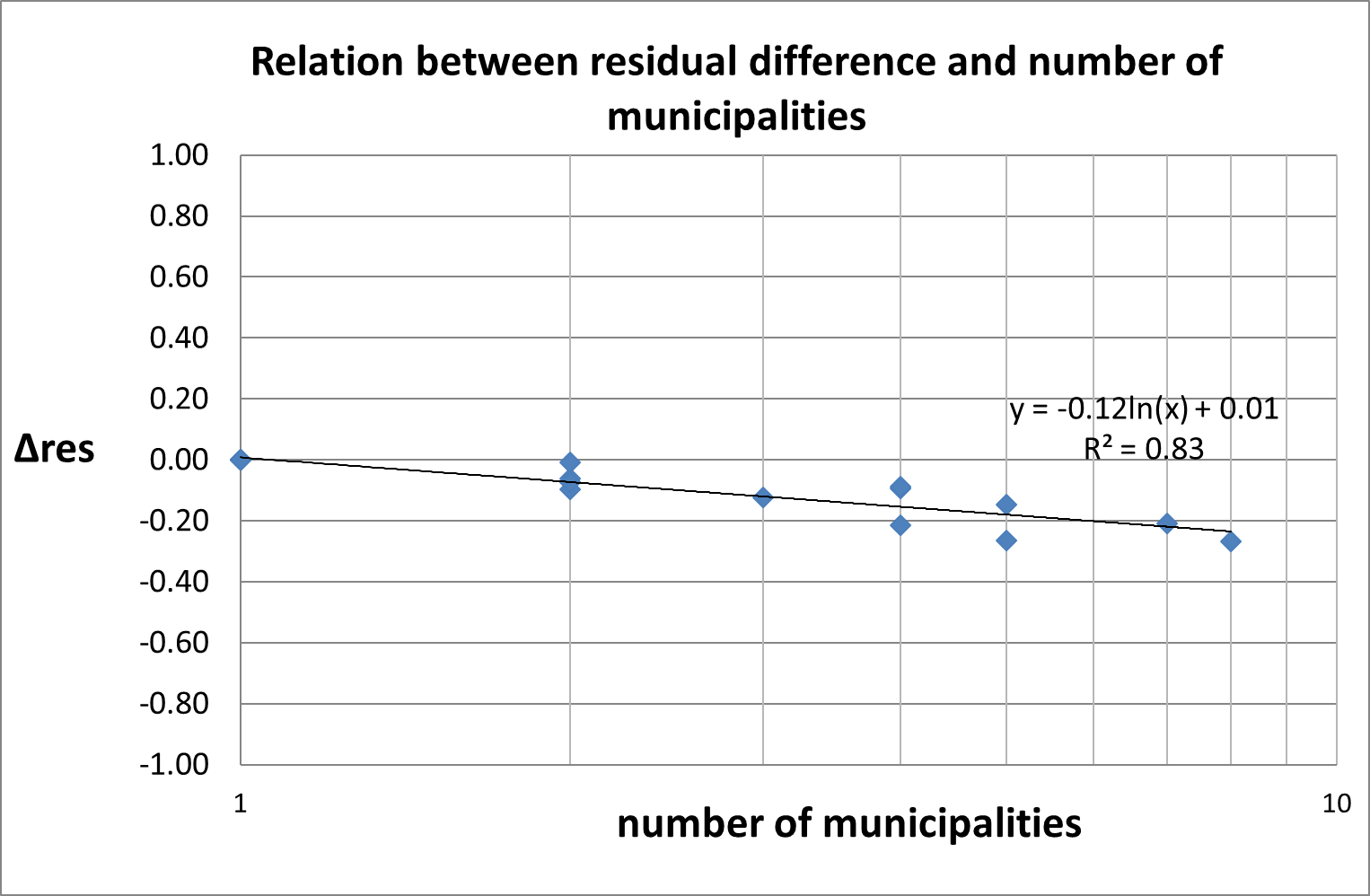


**Fig A2.5 Relation between residual difference and number of municipalities in urban agglomerations.**

We find that *∆res* has a negative, logarithmic correlation with the number of municipalities:

*∆res*=-0.12ln(*n*) + 0.01.

This measurement shows that the more municipalities in an urban agglomeration of a certain population size, the larger the negative effect in terms of GUP as compared to a one-governance city with the same population size. This finding is in line with the results of a recent OECD study [3].

References

1. van Raan AFJ, van der Meulen G, Goedhart W (2016). Urban Scaling of Cities in the Netherlands. *PLoS ONE* 11(1): e0146775.

2. Marlet G, van Woerkens C (2017). *Atlas voor Gemeenten 2017*. Nijmegen: VOC Uitgevers. See also <https://www.atlasvoorgemeenten.nl/de-atlas/de-atlas>.

3. Ahrend, R., Farchy E., Kaplanis, I., Lembcke, A. (2014). *What Makes Cities More Productive? Evidence on the Role of Urban Governance from Five OECD Countries*. OECD Regional Development Working Papers, No. 2014/05. Paris: OECD Publishing.
