# Supplementary material for: Urban scaling, geography, centrality: Relation with local government structures
Source: PLoS One. 2020 Sep 4;15(9):e0238418. doi: 10.1371/journal.pone.0238418 (PMC7473566; doi:10.1371/journal.pone.0238418)
Supplement: S3 Appendix — (DOCX) [file pone.0238418.s005.docx]

S3 File Appendix 3: Zipf-distribution

Does the distribution of population over municipalities with a Kreis follow a power law? We find a wide variety in power-law exponents and in the statistical significance of these power laws. As examples we show in Fig A3.1, left panel, the Zipf-distribution of four Kreise based on the largest six municipalities within the Kreis. We notice that in these cases there is a power-law distribution with high significance, but the exponent varies between -0.43 and -1.18. As discussed earlier, in the case of a flatter distribution (exponent -0.43, Rhein-Neckar Kreis) we have a more polycentric Kreis, and in the case of a steeper distribution (exponent -1.18, Garmisch-Partenkirchen) the Kreis is more monocentric. An example of a more extreme case is Kreis Kelheim, see Fig A3.1, right panel.


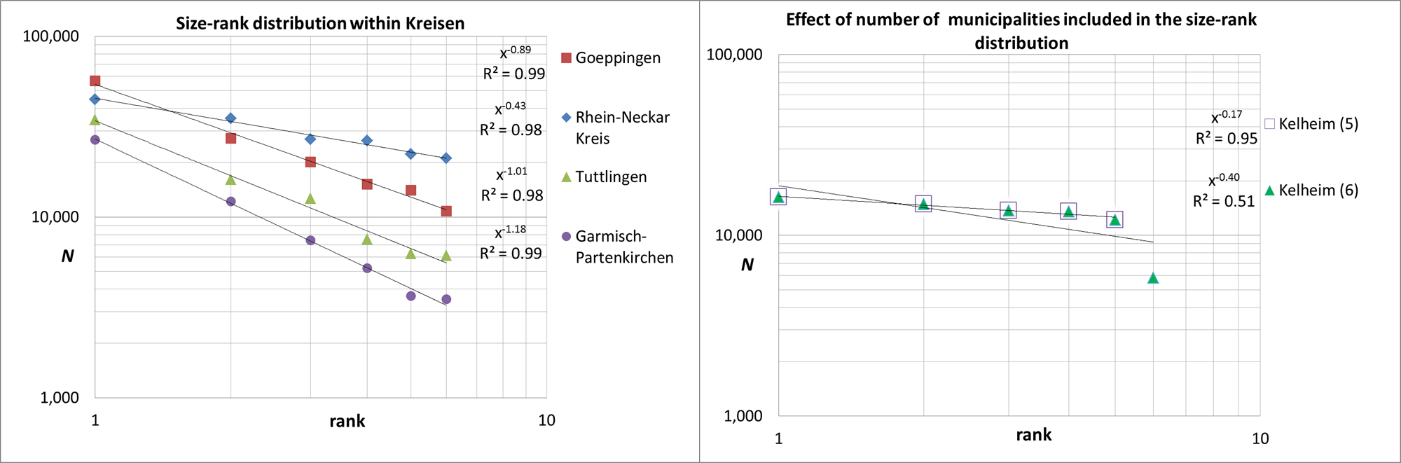


**Fig A3.1. Left panel: examples of size-rank distribution within Kreise, size distribution follows power law. Right pane;: example of the problem in measuring the size-rank distribution related to the number of municipalities (5 versus 6) included in the size-rank distribution.**

This shows the typical problem in the determination of the Zipf exponent: if we include the five largest municipalities of this Kreis in the measurement, we find with high significance an exponent -0.17. But if we include the next municipality the exponent is -0.40 with, as can be expected, a much lower significance.
